# The Gemin Associates of Survival Motor Neuron Are Required for Motor Function in *Drosophila*

**DOI:** 10.1371/journal.pone.0083878

**Published:** 2013-12-31

**Authors:** Rebecca Borg, Ruben J. Cauchi

**Affiliations:** 1 Department of Physiology and Biochemistry, University of Malta, Msida, Malta G.C; 2 Department of Applied Biomedical Sciences, University of Malta, Msida, Malta G.C; Imperial College London, United Kingdom

## Abstract

Membership of the survival motor neuron (SMN) complex extends to nine factors, including the SMN protein, the product of the spinal muscular atrophy (SMA) disease gene, Gemins 2–8 and Unrip. The best-characterised function of this macromolecular machine is the assembly of the Sm-class of uridine-rich small nuclear ribonucleoprotein (snRNP) particles and each SMN complex member has a key role during this process. So far, however, only little is known about the function of the individual Gemin components *in vivo*. Here, we make use of the *Drosophila* model organism to uncover loss-of-function phenotypes of Gemin2, Gemin3 and Gemin5, which together with SMN form the minimalistic fly SMN complex. We show that ectopic overexpression of the dead helicase Gem3^ΔN^ mutant or knockdown of Gemin3 result in similar motor phenotypes, when restricted to muscle, and in combination cause lethality, hence suggesting that Gem3^ΔN^ overexpression mimics a loss-of-function. Based on the localisation pattern of Gem3^ΔN^, we predict that the nucleus is the primary site of the antimorphic or dominant-negative mechanism of Gem3^ΔN^-mediated interference. Interestingly, phenotypes induced by human SMN overexpression in *Drosophila* exhibit similarities to those induced by overexpression of Gem3^ΔN^. Through enhanced knockdown we also uncover a requirement of Gemin2, Gemin3 and Gemin5 for viability and motor behaviour, including locomotion as well as flight, in muscle. Notably, in the case of Gemin3 and Gemin5, such function also depends on adequate levels of the respective protein in neurons. Overall, these findings lead us to speculate that absence of any one member is sufficient to arrest the SMN-Gemins complex function in a nucleocentric pathway, which is critical for motor function *in vivo*.

## Introduction

Spinal muscular atrophy (SMA) counts among an expanding ensemble of motor neuron degenerative disorders resulting from mutations in genes that encode proteins with crucial roles in RNA metabolism. The correct handling and processing of RNA from its transcription to eventual translation is emerging as key to the function and survival of the motor unit, relative to other tissues, for reasons as yet unclear [1], [2], [3], [4]. A common genetic cause of infant death, SMA is recessively inherited and is characterised by loss of lower motor neurons as well as progressive muscle weakness and wasting [5]. In the majority of cases, this devastating disorder is the result of low levels of the ubiquitously-expressed survival motor neuron (SMN) protein [6]. SMN forms oligomers [7],[8],[9] that associate with a core group of proteins including Gemin2-Gemin8 [10], [11],[12],[13],[14],[15],[16],[17],[18] and Unrip [19], [20] to form the large macromolecular SMN complex (reviewed in [21]).

Across metazoa, the SMN complex is particularly concentrated in several prominent cellular bodies, called gems within the nucleus [22], [23] or U bodies if they dot the perinuclear periphery in the cytoplasm [24], [25]. The exact function of these organelles is not yet known though they share several characteristics (reviewed in [21]), including crosstalk with other compartment-specific organelles [22], [26], [27] and response to metabolic changes in the cell [22], [28]. The best-characterised function of the SMN complex revolves around the biogenesis of uridine-rich small nuclear ribonucleoproteins (snRNPs) which are the central elements of the spliceosome and, hence, crucial for yielding mature spliced mRNAs [6], [21], [29]. All SMN complex members bar Unrip are indispensable for the uploading of a heptameric ring of Sm proteins onto small nuclear RNAs (snRNAs) to form the core structure of Sm-class snRNPs [16], [20], [30], [31], [32].

Details about the role of each member of the SMN complex in the assembly process are scant. Sm proteins are thought to be recognised by Gemin2, which wraps itself around a pentamer formed of Sm D1/D2/E/F/G to contact all five Sm proteins. Importantly, the N-terminal tail of Gemin2 reaches into the snRNA-binding pocket on the Sm pentamer to occlude RNA binding, presumably until the delivery of bona fide RNA substrates, snRNAs [33]. Gemin5 is the factor that identifies snRNAs through the stringent recognition of a code formed of sequences as well as structural motifs [34]. Newly exported snRNAs are captured by SMN complex-independent Gemin5 via its N-terminal WD-repeat domain [35], which then docks into the SMN complex, most probably proximate to Gemin2, to deliver its load for Sm core assembly [36]. Gemin3 is a functional DEAD-box RNA helicase [37], and might be necessary for chaperoning RNA and, eventually, RNP complexes during the assembly reaction with the intimately-associated Gemin4, possibly acting as a co-factor for such activities. Regarding SMN and considering its oligomeric nature within the cellular milieu, it is highly probable that SMN, aided by other SMN complex components, acts as a scaffold on which more than one assembly reaction are occurring simultaneously.

Whether a decreased capacity to assemble snRNPs and, consequently, defective splicing, accounts for the selective neuromuscular phenotype in SMA is still a passionately debatable topic in the field. An alternative view proposes that SMN has a motor neuron-specific function that is secondary to its housekeeping activities, and it is loss of this critical function in the context of sufficient snRNP assembly that might explain the profound tissue-specificity observed in SMA patients [6],[38]. However, it is still an open question whether all or select members of the SMN complex participate in such a non-canonical function if at all present. In part allured by the presence of a functional minimalistic complex composed of only SMN, Gemin2, Gemin3 and Gemin5 [21], [25], [39], [40], [41], we have exploited *Drosophila* as a genetic systems model to investigate the workings of the SMN complex *in vivo* and, hence, shed light on these conundrums. Recently, we reported that *Gemin3* null larval mutants exhibit phenotypes that were strikingly similar to those observed for *Smn* mutants including a decline in mobility and defects at the neuromuscular junction [40], [42]. Interestingly, overexpression of a truncated Gemin3 mutant lacking the helicase core (Gem3^ΔN^) or Gemin3 knockdown, in muscle but not neuronal tissue, both have a drastic impact on adult viability. Remarkably, when pan-muscular overexpression is milder, Gem3^ΔN^ confers flight defects and flight muscle atrophy, a phenotype reminiscent of that observed in a hypomorphic *Smn* mutant (*Smn^E33^*) reported to have low SMN protein levels in adult flight muscles [40], [43].

In the present study, we first sought to investigate the dominant-negative or antimorphic mechanism responsible for the overexpression phenotype of Gem3^ΔN^. We show that loss-of-function through knockdown of Gemin3 in muscle tissue gives rise to similar motor phenotypes including climbing defects and loss of flight. Notably, Gemin3 knockdown and overexpression of Gem3^ΔN^, in combination but not singularly, within either muscle or neuronal tissue of the same organism is lethal. These findings indicate that Gem3^ΔN^ mimics a loss-of-function by presumably interfering at some level with the activity of the Gemin3 protein or its complex. Tagging Gem3^ΔN^ with GFP allowed us to delve into the sub-cellular location of such interference, which we predict to be predominantly nuclear and similar in certain aspects to that of human SMN, which was also previously reported to be antimorphic [44]. Through an enhanced knockdown screen in diverse spatial and temporal patterns, we also show that Gemin3 is essential for viability and flight not only in muscle but also in the brain. Intriguingly, we uncover a similar requirement for Gemin5, hence its enhanced knockdown in either brain or muscle tissue gives rise to viability and flight defects. We finally tackle the function of Gemin2, which we also find to be important in the motor unit for motor behaviour including flight. Placed within the context of previous studies, our results lead us to hypothesise about the presence of a common nucleocentric pathway or process for SMN and its Gemin associates that is critical for motor function *in vivo*.

## Results

### Gemin3 Knockdown in Muscle Tissue Results in Adult Age-progressive Climbing Defects

According to Muller’s 1932 classical classification of mutations, an *antimorph* is defined as a mutant allele that antagonises its co-expressed wild-type gene product, thereby reducing its functional activity [45], [46]. In an era where the molecular basis of Muller’s antimorphs could be comprehended, Herskowitz [47] coined the term *dominant-negative* which has been used synonymously with antimorph ever since. Helicase-inactivating mutations can exert a spectrum of dominant-negative phenotypes [48]. DEAD-box RNA helicase Gemin3 is present in all metazoans and high levels of amino acid conservation are observed within the N-terminal helicase motifs [40], [49]. We recently reported on the generation of a truncated version of Gemin3 lacking the helicase domains, Gem3^ΔN^ (**FIG S1A**). Whereas expression of this catalytically inactive mutant fails to rescue the lethality associated with a *Gemin3* null background, its ubiquitous or pan-muscular-specific overexpression in wild-type organisms, is lethal ( [40] and data not shown). A milder pan-muscular overexpression is however associated with a flightless phenotype [40].

**Figure 1:**
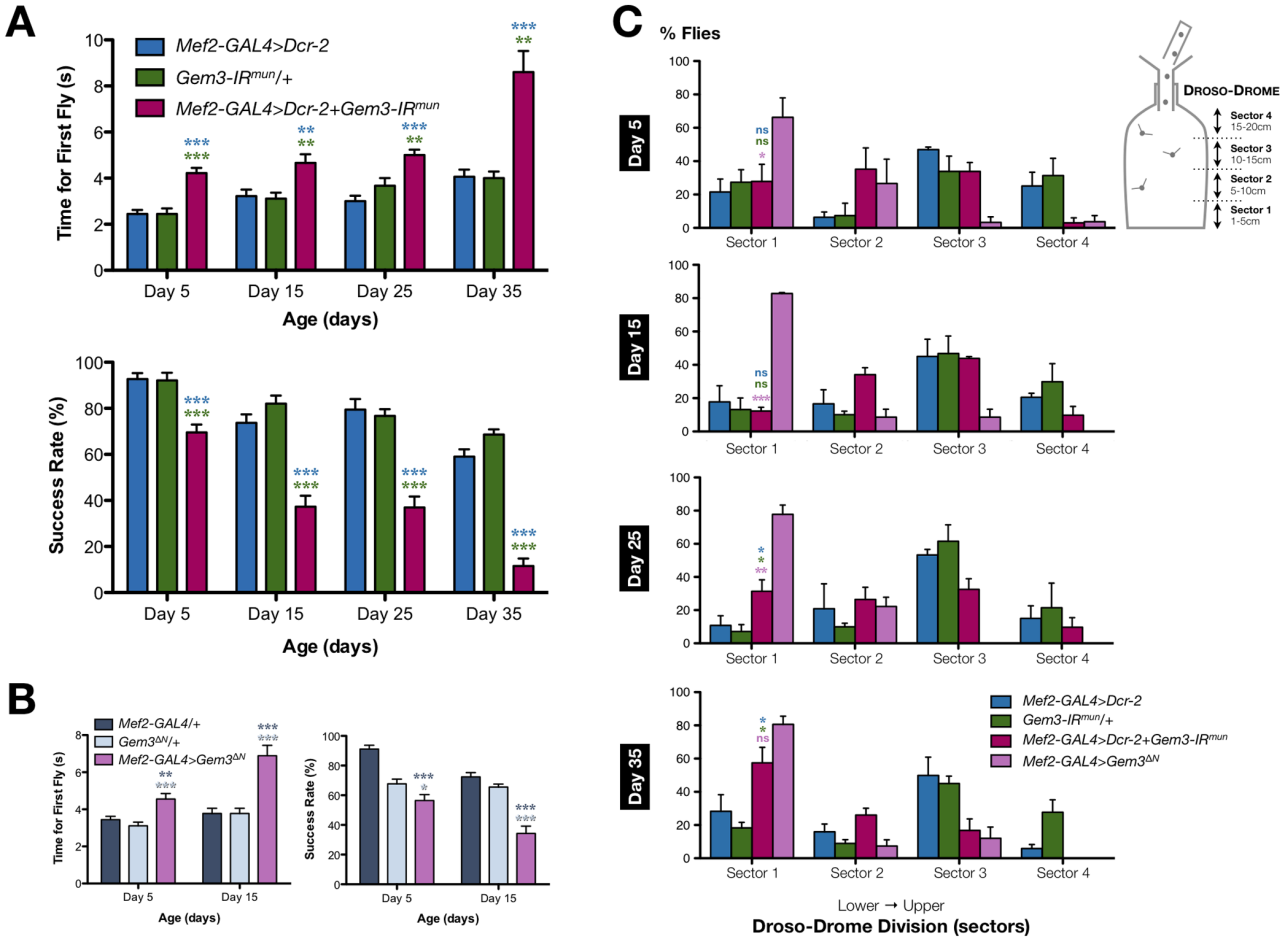
Pan-muscular Gemin3 knockdown or Gem3^ΔN^ overexpression gives rise to identical motor phenotypes. (**A**) Compared to controls (*Mef2-GAL4>Dcr-2* and *Gem3-IR^mun^/+*), the time taken for the first fly to climb 8 cm increases progressively over the course of 35 days in sample populations having reduced levels of Gemin3 in muscles (*Mef2-GAL4>Dcr-2+Gem3-IR^mun^*). The latter genotype also exhibits an age-dependent progressive decline in the total percentage of flies that successfully reached the 8 cm mark within 10 seconds when compared to the same controls. (**B**) An identical progressive change in climbing behaviour is observed between flies with pan-muscular Gem3^ΔN^ overexpression (*Mef2-GAL4>Gem3* ^Δ*N*^) and the uncombined driver (*Mef2-GAL4/+*) or transgene (*Gem3* ^Δ*N*^ */+*) controls. The day 25 and day 35 time points were excluded from the chart due to a lack of difference with the driver control (*Mef2-GAL4/+*). (**C)** Flight behaviour was determined using the Droso-Drome, in which the height a fly falls determines its flight performance. Fliers tend to stick to the upper sectors whereas non-fliers drop to sector 1, the lowest sector. On day 5 and day 15 post-eclosion, the percentage of flight-impaired organisms in the Gemin3 knockdown population (*Mef2-GAL4>Dcr-2+Gem3-IR^mun^*) is not significantly different to that of controls. This situation changes on the day 25 and day 35 time points whereby a significant difference is obvious. At day 35, the percentage of flightless flies in the *Mef2-GAL4>Dcr-2+Gem3-IR^mun^* population are in the majority such that significant differences with flies overexpressing Gem3^ΔN^ (*Mef2-GAL4>Gem3* ^Δ*N*^) do not persist at this time point. Data presented are the mean ± S.E.M. and statistical significance was determined for differences between the *Mef2-GAL4>Dcr-2+Gem3-IR^mun^* (A, C) or *Mef2-GAL4>Gem3* ^Δ*N*^ (B) genotype and other genotypes, which are indicated by the respective colour. For all data, ns = not significant, *p<0.05, **p<0.01, and ***p<0.001.

We attempted to investigate the mechanism through which overexpression of the Gem3^ΔN^ mutant confers a dominant-negative or antimorphic phenotype. First, we asked whether loss of Gemin3 function through RNA interference (RNAi)-mediated knockdown gives rise to an identical phenotype. To this end, making use of the versatile GAL4/upstream activation sequence (UAS) system (reviewed in [50]), we expressed an inducible RNAi transgene targeting Gemin3 (*Gem3-IR^mun^;* **FIG S1A**) in muscle tissues using the pan-muscular *Mef2-GAL4* driver. The specificity of this transgene was demonstrated previously [40], and co-expression of *Dicer-2* (*Dcr-2*) was also performed in order to enhance knockdown efficiency [51]. Flies were then subjected to a climbing assay at 5, 15, 25 and 35 days post-eclosion. The first parameter measured was the time taken for the first fly in the sampled population to climb a height of 8 cm. At all days measured, the first fly in the *Mef2-GAL4>Dcr-2+Gem3-IR^mun^* fly population took, on average, significantly longer to reach the target height compared to controls (**FIG. 1A**). To better reflect the climbing ability of all flies in a sampled population, we counted the number of flies that reached a height of 8 cm by 10 seconds to determine the second parameter, namely the climbing success rate. Similar to the data on the time for first fly, flies with reduced levels of Gemin3 had a significantly lower average success rate at all time points when compared to controls (**FIG. 1A**). Furthermore, their success rate showed an age-dependent progressive decline in climbing ability; hence, the worst result was obtained on the last time point measured, namely, day 35 post-eclosion. Notably, these results are similar to those obtained by flies with a GAL4/UAS-mediated pan-muscular overexpression of Gem3^ΔN^. Indeed, compared to controls, top performers in the *Mef2-GAL4>Gem3* ^Δ*N*^ population took progressively longer, on average, to reach the target height in contrast to controls. The average climbing success rate in this population was also significantly lower than that of controls, and showed an age-dependent deterioration (**FIG. 1B**). Therefore, Gemin3 attenuation in muscle leads to mobility deficits that are similar to those resulting from ectopic Gem3^ΔN^ overexpression in the same tissue.

### Gemin3 Knockdown in Muscle Tissue Leads to Age-dependent Flight Defects

Metazoans invest substantially in muscles that power movement and this is especially true in insects where flight muscles alone can make up as much as 65% of the total body mass [52]. Some insects including *Drosophila* evolved a remarkable muscle that is capable of generating high power at high frequency, hence generating sufficient force to offset gravity [53]. At the same time, there remains substantial conservation from insects to mammals in the basic cell and developmental biology of muscles [54], [55]. In addition to a powerful yet lightweight engine supplied by flight muscles, an animal capable of active flight must also possess wings that are capable of generating sufficient aerodynamic forces as well as a control system, in which motor neurons are pivotal, to keep the organism from plummeting to the ground [53]. Deficiency of a protein with an indispensible function in either or all three elements – muscles, wings and sensory-motor circuit – is predicted to have a negative impact on flight.

In this context, besides climbing ability, we wanted to determine the flight performance in a sampled population of flies. To this end, we developed the Droso-Drome, a device that enables systematic quantification of flight defects based on the sensitive ‘cylinder drop assay’ described previously [56]. Basically, the height a fly falls determines its flight capability, with fliers able to hold onto the walls of upper sectors whereas flight defective organisms fall to the lower sectors. We observe that when subjected to this assay, as we previously reported [40], the majority of flies with pan-muscular overexpression of Gem3^ΔN^ are flightless on day 5 post-eclosion, the earliest time point measured, and, hence, 66% of the flies fall straight to sector 1, the lowest sector (**FIG. 1C**). At day 5 and 15 post-eclosion, compared to driver- (*Mef2-GAL4>Dcr-*2) and transgene-only (*Gem3-IR^mun^/+*) controls, we did not observe large differences in the fly population induced to have pan-muscular knockdown of Gemin3 (*Mef2-GAL4>Dcr-2+Gem3-IR^mun^*). Furthermore, at these time points, such flies behave differently from the *Mef2-GAL4>Gem3* ^Δ*N*^ fly population indicating that they fly well. Although the latter difference remained, we started to notice differences in the percentage of non-fliers between Gemin3 knockdown flies (31%) and controls (*Mef2-GAL4>Dcr-2*, 11% and *Gem3-IR^mun^/+*, 7%) on the day 25 time point. However, a dramatic difference was observed on day 35 post-eclosion whereby the distribution of *Mef2-GAL4>Dcr-2+Gem3-IR^mun^* flies was significantly different to that exhibited by the control populations and similar to that of *Mef2-GAL4>Gem3* ^Δ*N*^ flies. In this regard, at this time point, flight-defective organisms that tumbled straight to sector 1 are in the majority within the *Mef2-GAL4>Dcr-2+Gem3-IR^mun^* (57%) and *Mef2-GAL4>Gem3* ^Δ*N*^ (80%) populations (**FIG. 1C**). This indicates that similar to Gem3^ΔN^ overexpression, Gemin3 knockdown leads to a flight defective phenotype, albeit age-dependent.

### Gemin3 knockdown And Gem3^ΔN^ Overexpression, in Combination, Lead to Lethality

**Figure 2:**
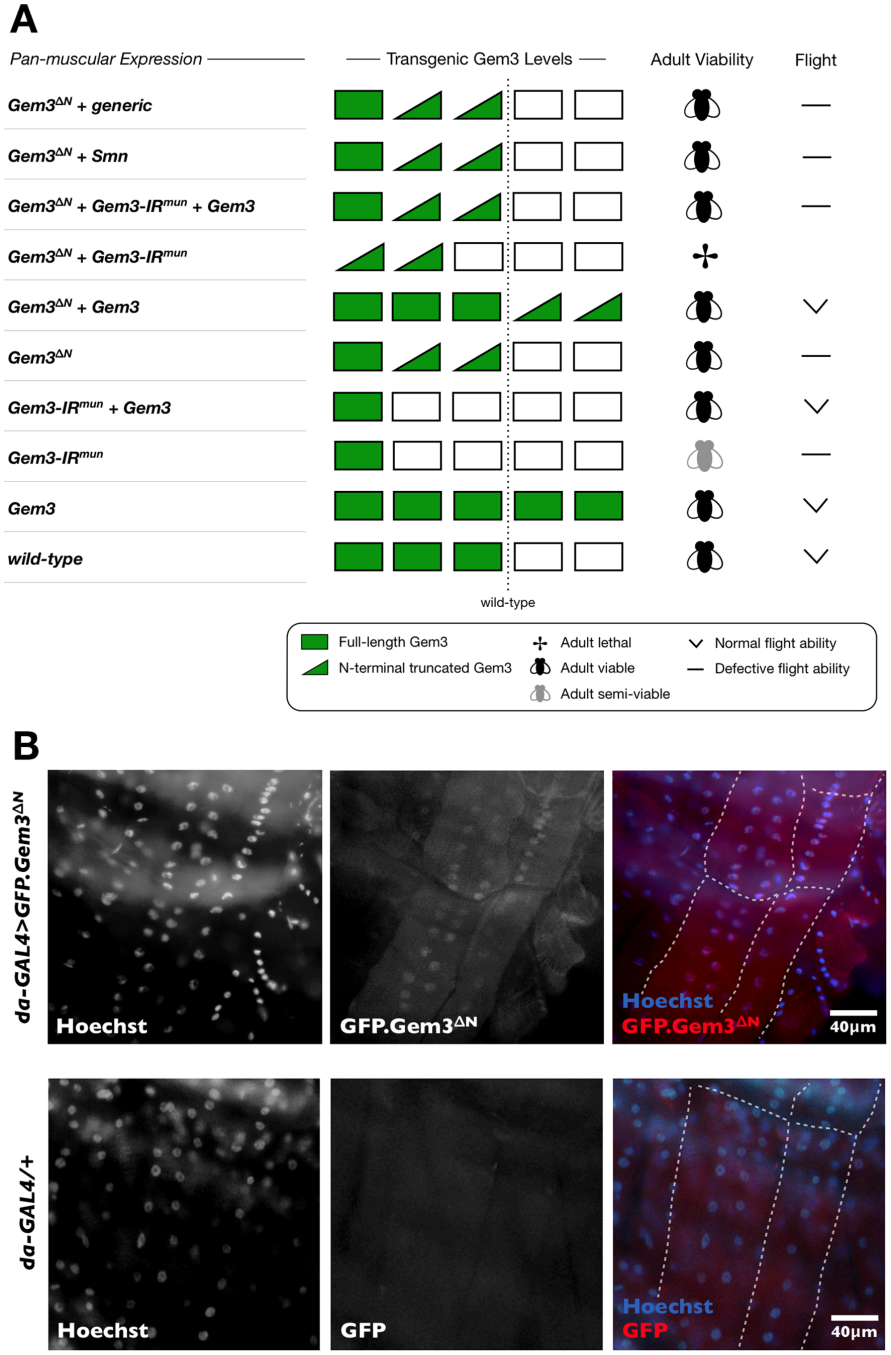
Gem3^ΔN^ in combination with Gemin3 knockdown is lethal and its localisation pattern is predominantly nuclear. (**A**) Perturbation of endogenous Gemin3 in wild-type muscle and its consequences on adult viability and flight behaviour. *Bottom* to *Top*. Overexpression of full-length Gemin3 has no repercussions. Reduced levels of Gemin3 induced through the expression of *Gem3-IR^mun^* is however associated with a progressive age-dependent decline in flight and adult viability, which can be rescued by overexpression of full-length Gemin3. Overexpression of Gem3^ΔN^ has no effect on adult viability but has a drastic impact on flight straight after eclosion, which again can be rescued by overexpression of full-length Gemin3. Gemin3 knockdown in combination with Gem3^ΔN^ overexpression results in lethality. Overexpression of full-length Gemin3 in this background rescues the associated lethality but not the flight defects. SMN overexpression in a Gem3^ΔN^ background has no influence on the anomalous flight behaviour (assessment at day 5 post-eclosion) associated with this genotype. Co-expression of an unrelated transgene (generic) with *Gem3* ^Δ*N*^ still results in a flightless phenotype thereby excluding the possibility of a GAL4 dilution effect resulting from multiple UAS-constructs in the same organism. Pan-muscular expression was driven by *Mef2-GAL4* and experiments were performed at 25°C. Protein levels of transgenic Gemin3 are indicated by bar strength. (**B**) Subcellular localisation of GFP.Gem3^ΔN^ in *Drosophila* larval muscles (delineated by a dashed white outline). In flies overexpressing GFP.Gem3^ΔN^, the GFP signal is ubiquitous but predominantly nuclear as it co-localises with Hoechst-stained nuclei. This subcellular expression pattern was not visible in the driver control (*da-GAL4/+*), hence, excluding the possibility that the result is due to the non-specific reactivity of the primary and/or secondary antibodies used to detect GFP.

If Gem3^ΔN^ overexpression mimics a loss-of-function, we predicted that Gem3^ΔN^ overexpression in combination with Gemin3 knockdown within the same organism would lead to a phenotype that is more severe than that resulting when both genetic manipulations are applied singularly. To test this hypothesis, we generated organisms with an *Mef2*-GAL4-driven Gemin3 knockdown and Gem3^ΔN^ overexpression. Remarkably, in combination within muscle, both genetic manipulations result in lethality or total loss of adult viability (**FIG. 2A**). This phenotype contrasts heavily with that observed by both elements, distinctively. In this regard, pan-muscular Gem3^ΔN^ overexpression alone has no effect on adult viability though flight is impaired (**FIG. 2A** and above). Furthermore, RNAi-induced depletion of Gemin3 levels in muscle, by itself, is not lethal, and only results in an age-dependent progressive decline in adult survival (day 5, 100%; day 15, 75%; day 25, 66%; and day 35, 62%) as well as flight ability (**FIG. 2A** and above).

Although overexpression of full-length Gemin3 in muscle of wild-type flies has no effect on adult viability or flight ability, transgene levels were adequate to rescue the flight defects associated with RNAi-mediated Gemin3 knockdown or Gem3^ΔN^ overexpression. Furthermore, when Gemin3 was added back to a background having both genetic manipulations, transgenic protein levels were capable of rescuing lethality but they were not enough to revert flight performance to normal (**FIG. 2A**). Finally, we show that overexpression of SMN in a Gem3^ΔN^ background does not rescue the associated flight defects indicating that these do not arise due to downstream events that result in deficient SMN function. Taken together, these findings first confirm that Gem3^ΔN^ overexpression mimics a strong loss of Gemin3 function. Second, a drastic reduction in Gemin3 function within muscle results in lethality whereas low levels are not enough to sustain normal motor behaviour. Third, Gemin3 has an indispensable motor function that cannot be adequately fulfilled by a simple expansion in the functional capacity of SMN.

### Gem3^ΔN^ Localises Primarily to the Nucleus

Epitope tagging or the fusion of a known epitope to a recombinant protein has revolutionised the characterisation of proteins and their mutant versions, especially those with low immunogenicity [57]. Taking advantage of this technique, we engineered a GFP-tagged Gem3^ΔN^ fusion protein with the goal of determining the sub-cellular location where the mutant dead helicase protein antagonises or interferes with the function of the endogenous Gemin3 protein. We note that overexpression of the GFP.Gem3^ΔN^ fusion protein in muscle induces the same flightless phenotype observed by its tag-free counterpart, hence, indicating that the insertion of the epitope tag did not alter the antagonistic effects of Gem3^ΔN^ (data not shown). Importantly, on staining for GFP, we observed that although GFP.Gem3^ΔN^ is ubiquitously present throughout muscles, its localisation pattern is predominantly nuclear (**FIG. 2B**). In contrast to full-length wild-type Gemin3, which localises to several intensely-stained foci (gems) in the nucleus [23], Gem3^ΔN^ gives a diffuse nuclear staining pattern.

### Phenotypes Resulting from Ectopic Human SMN Overexpression are Similar to those Resulting from Gem3^ΔN^-mediated Interference

**Figure 3:**
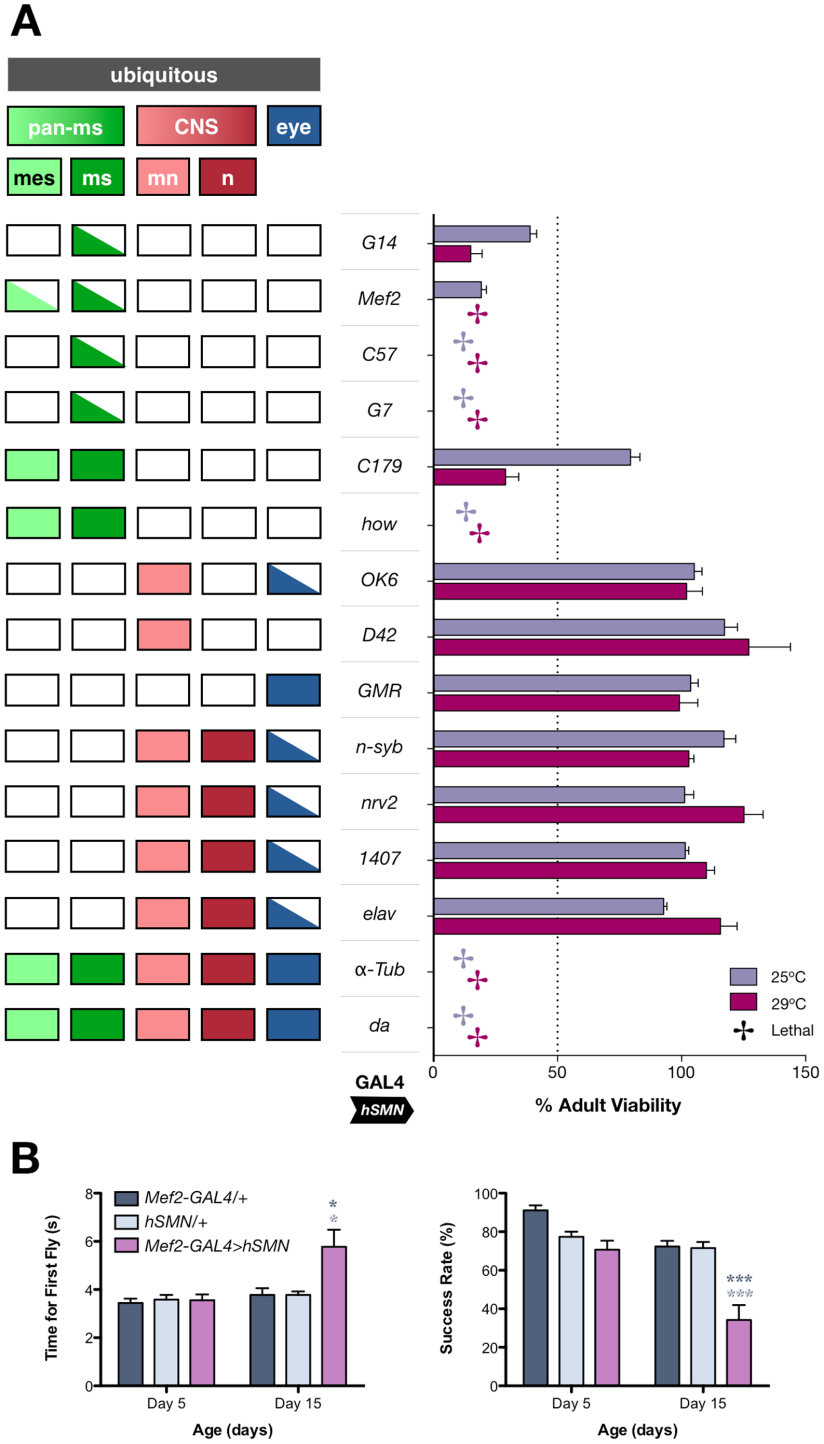
Ectopic overexpression of hSMN in wild-type organisms affects viability and motor behaviour when restricted to muscle. (**A**) Ubiquitous overexpression of hSMN in wild-type flies via *α-Tub-*GAL4 or *da-*GAL4 is lethal. Restricting hSMN overexpression to mesoderm and larval muscles (via *how-*GAL4, *C179-*GAL4 or *Mef2-*GAL4) or larval muscles alone (via *G7-*GAL4, *C57-*GAL4 and *G14-*GAL4), leads to lethality or a drastic reduction in viability. Fly viability remains unaffected when hSMN is overexpressed in all CNS neurons (via *elav-*GAL4, *1407-*GAL4, *nrv2-*GAL4 or *n-syb-*GAL4) or when it is restricted only to motor neurons (via *D42-*GAL4 or *OK6-*GAL4) or eye (*GMR-*GAL4). Bar chart (right panel) show adult fly viability assayed at 25°C and 29°C, the latter resulting in enhanced GAL4 activity. Individual bars represent the mean viability ± S.E.M. of at least 4 independent experiments. Left panel shows the tissue expression pattern of all GAL4 drivers utilised. Abbreviations: *pan-ms*, pan-muscular; *mes*, mesoderm; *ms*, larval muscles; *mn*, motor neurons; *n*, all CNS neurons except motor neurons. (B) The climbing behaviour of flies with pan-muscular hSMN overexpression (*Mef2-GAL4>hSMN*) is significantly altered by day 15 post-eclosion compared to the control sample populations (*Mef2-GAL4/+* or *hSMN/+*). Data presented are the mean ± S.E.M. and statistical significance (*p<0.05, and ***p<0.001) was determined for differences between the *Mef2-GAL4>hSMN* genotype and control genotypes, which are denoted by the respective colour.

**Figure 4:**
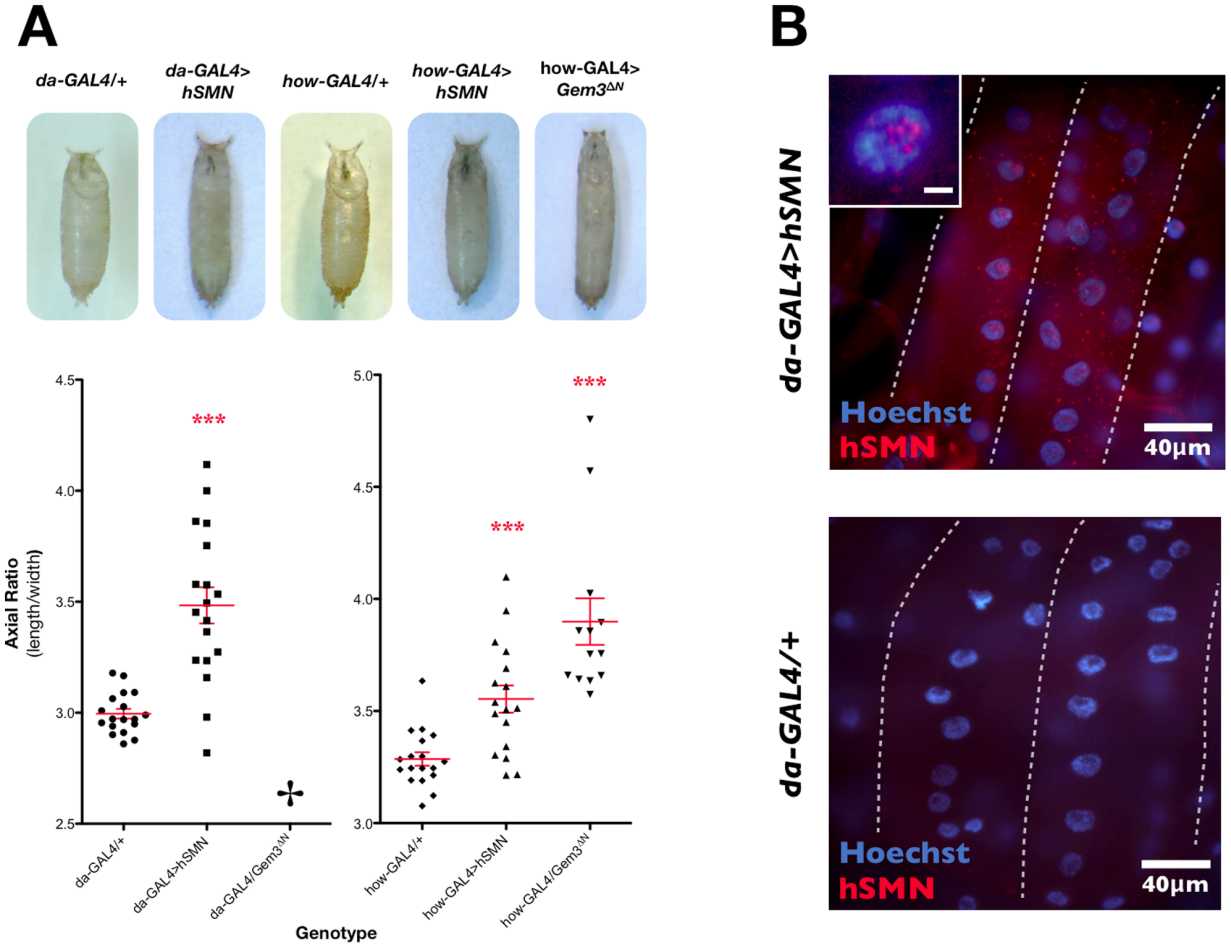
Overexpression of hSMN in wild-type organisms interferes with the normal puparial axial ratio in a similar manner to Gem3^ΔN^, and induces the formation of spherical aggregates. (A) *Top*, Puparia of flies with ubiquitous overexpression of hSMN (*da-GAL4>hSMN*), pan-muscular overexpression of hSMN (*how-GAL4>hSMN*) or Gem3^ΔN^ (*how-GAL4>Gem3* ^Δ*N*^), and their respective GAL4 driver controls (*da-GAL4/+* or *how-GAL4/+*). *Bottom*, Chart displaying the axial ratios for puparia of the indicated genotype. Ubiquitous or pan-muscular overexpression of hSMN results in significantly larger puparial axial ratios. Although ubiquitous overexpression of Gem3^ΔN^ results in lethality prior to puparium formation, its pan-muscular overexpression results in puparia with a significantly large axial ratio. The mean is marked by a horizontal line running through the data points and error bars are ± S.E.M. (***p<0.001). (B) Larval muscles (delineated by dashed white outline) showing the formation of hSMN-positive spherical aggregates of variable size in the cytoplasm and nucleus of flies with a ubiquitous ectopic overexpression of hSMN. Within the nucleus, these foci localise to the nucleolus, which is the region with low Hoechst reactivity (insert, scale bar = 5 µm). Aggregates were not visible in control (*da-GAL4/+*) tissues subjected to the same immunostaining reaction, thereby excluding the possibility of non-specific reactivity.

Ectopic overexpression of the human SMN (hSMN) protein in wild-type flies has been reported to antagonise endogenous *Drosophila* SMN (dSMN) function. The presumed mechanism responsible for the resulting dominant-negative phenotype has been ascribed to the binding of hSMN to endogenous dSMN, eventually sequestering the latter into non-functional hSMN/dSMN hetero-oligomers [44]. Ubiquitous expression of hSMN but not dSMN in an *Smn* null background (*Smn^X7^/Smn^X7^*) fails to rescue the lethality associated with this genetic background (data not shown), most probably the result of its divergence from dSMN. To shed further light on the mechanism of action of Gem3^ΔN^, we probed into the similarities and differences between the downstream events resulting from hSMN or Gem3^ΔN^ overexpression. To this end, we noted that similar to that previously reported for Gem3^ΔN^ [40], pan-muscular but not pan-neuronal overexpression of hSMN induces lethality or reduced adult viability (**FIG. 3A**). Furthermore, similar to those with Gem3^ΔN^ overexpression (see above), flies with hSMN overexpression in muscle exhibited climbing defects (**FIG. 3B**). Despite these observations, and in contrast to Gem3^ΔN^, we did not identify any large differences in the flight behaviour between flies with pan-muscular hSMN overexpression and their respective controls (**FIG S2**). However, overexpression of Gem3^ΔN^ or hSMN both resulted in an increase in puparial axial ratios (**FIG. 4A**), a phenotype described previously in *Gemin3* null mutants [40], and on modulation of *Drosophila* SMN protein levels [58], [59]. Interestingly, hSMN was detected in intensely-stained nuclear and, at times, cytoplasmic puncta within muscle tissue. Inside the nucleus, hSMN concentrates in multiple spherical puncta of different sizes, which are restricted to the nucleolus, which is devoid of Hoechst-labelled chromatin (**FIG. 4B**).

### Gemin3 is Required for Viability and Normal Motor Behaviour also in Neurons

Previously we demonstrated that a global reduction of Gemin3 function elicits a lethal phenotype, which can be recapitulated if Gemin3 is reduced solely in muscle but not neuronal lineages [40]. The conclusion we therefore reached was that in contrast to SMN which is essential in either tissue [42], [60], Gemin3 is required exclusively within muscle for survival and normal motor function. However, we did not exclude the possibility that the pan-neuronal GAL4 drivers used to induced RNAi-mediated Gemin3 knockdown were less potent and/or their expression started late during development in contrast to the GAL4 drivers with a pan-muscular expression pattern. Furthermore, RNAi might be less efficient in neurons compared to muscle. In this context, we could not rule out a neuronal function for Gemin3, and to this end, we devised several strategies aimed at providing a definitive answer.

**Figure 5:**
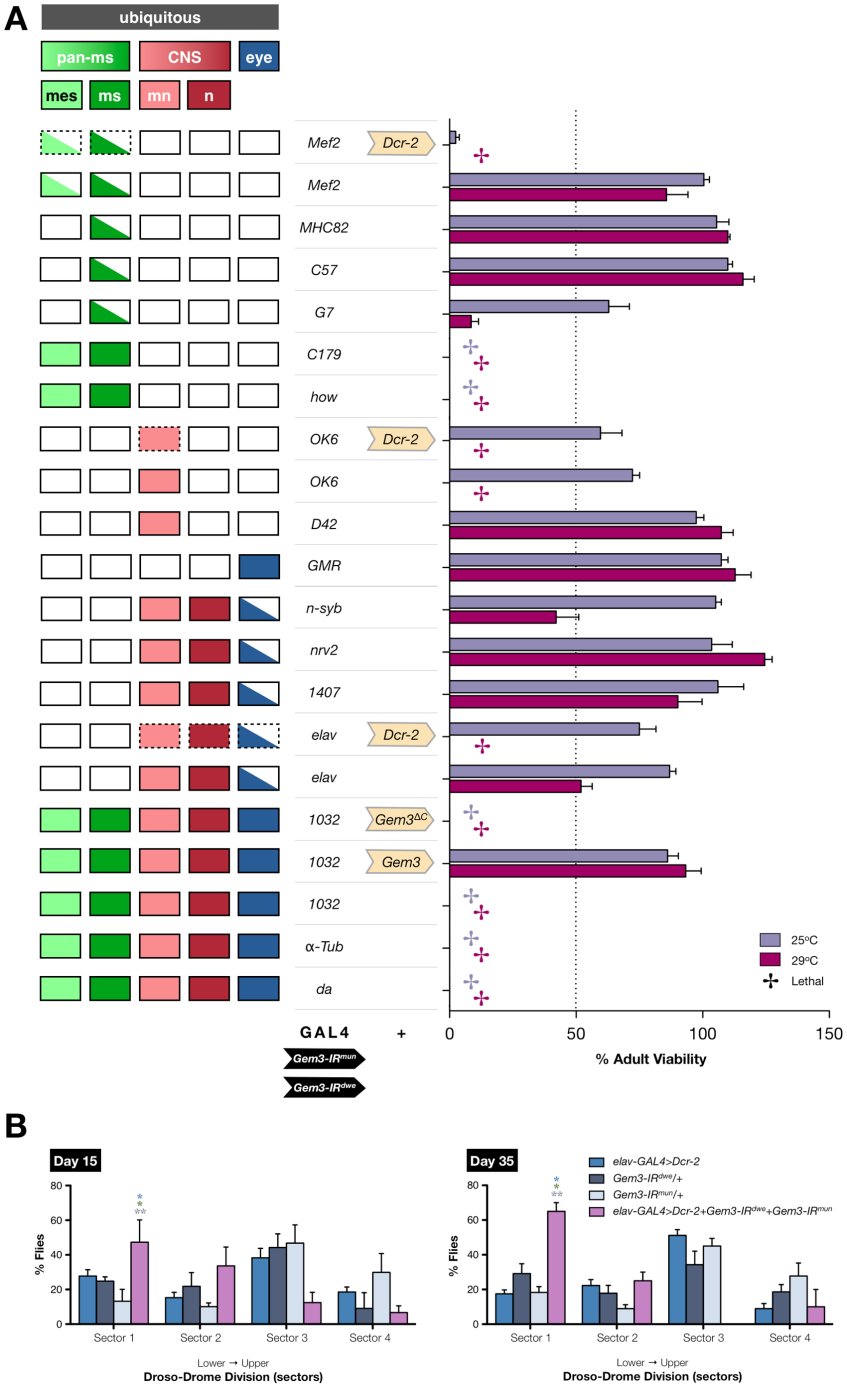
Enhanced Gemin3 knockdown in neuronal lineages leads to loss of adult viability and flight defects. (**A**) Enhanced Gemin3 knockdown involved the simultaneous application of two RNAi transgenes (*Gem3-IR^mun^+Gem3-IR^dwe^*) targeting Gemin3. Culture at a temperature of 29°C to allow for maximal GAL4 activity and/or Dicer-2 overexpression resulted in a further increase in RNAi potency. Gemin3 knockdown in all tissues via the *da*-GAL4, *α-Tub*-GAL4 or *1032*-GAL4 drivers results in lethality, which can be rescued on co-expression of a full-length (*Gem3*) but not a C-terminal truncated (*Gem3* ^Δ*C*^) *Gemin3* transgene, hence demonstrating RNAi specificity. When Gemin3 knockdown is restricted either to all muscle lineages (*how*-GAL4, *C179*-GAL4 or *Mef2*-GAL4) or only to the somatic musculature (*G7*-GAL4), loss of adult viability or complete lethality was observed, a phenotype that is similar or worse to that previously reported with a dose of one RNAi transgene [40]. Remarkably, we note that on pan-neuronal Gemin3 knockdown (via *n-syb*-GAL4 or *elav*-GAL4), we could also achieve a significant decrease in adult viability when culture temperature was raised to 29°C and, eventually, complete lethality on concomitant overexpression of Dicer-2. Knockdown exclusively in motor neurons was sufficient to markedly impact adult viability or cause lethality at an incubation temperature of 25°C and 29°C, respectively, with or without enhanced Dicer-2 levels. Compared to controls, enhanced knockdown of Gemin3 in the eyes via the *GMR*-GAL4 driver does not lead to a rough eye phenotype (data not shown). Note that, as expected, not all drivers with a similar tissue expression pattern (left panel) give an analogous result with one reason being that they might not drive strong GAL4 levels comparable to their effective counterparts. Bar chart (right panel) shows adult fly viability assayed at 25°C and 29°C. Individual bars represent the mean viability ± S.E.M. of at least 4 independent experiments. Abbreviations: *pan-ms*, pan-muscular; *mes*, mesoderm; *ms*, larval muscles; *mn*, motor neurons; *n*, all CNS neurons except motor neurons. (**B**) Flight behaviour of the population with a brain-restricted enhanced Gemin3 knockdown (*elav-GAL4>Dcr-2+Gem3-IR^dwe^+Gem3-IR^mu^* ^n^) is significantly different from that of controls starting at day 15 post-eclosion. In this regard, the number of non-fliers that drop straight to the lowest sector (sector 1) exhibits a progressive age-dependent increase that is not obvious in driver-only (*elav-GAL4>Dcr-*2) or responder-only (*Gem3-IR^dwe^/+* or *Gem3-IR^mun^/+*) control populations. Data presented are the mean ± S.E.M. and statistical significance was determined for differences between the *elav-GAL4>Dcr-2+Gem3-IR^dwe^+Gem3-IR^mun^* genotype and the control genotypes, which are indicated by the respective colour. For all data, ns = not significant, *p<0.05, **p<0.01, and ***p<0.001.

We first noticed that similar to that observed when restricted to muscle (above), Gem3^ΔN^ overexpression in combination with Gemin3 knockdown exclusively within neuronal tissues (via the pan-neuronal *elav*-GAL4 driver at an incubation temperature of 25°C) also induces lethality. This remarkable observation hinted at a critical role for Gemin3 in the central nervous system (CNS), which only becomes apparent following a severe reduction in function. To confirm this finding, we attempted at markedly attenuating the level of Gemin3 by increasing the dose of hairpin RNAs and, eventually, short interfering RNAs (siRNAs) targeting Gemin3. To this end, expression of two RNAi transgenic constructs (*Gem3-IR^mun^*+*Gem3-IR^dwe^*) in muscle had a similar or a worse effect to that reported previously using a single transgenic construct (**FIG. 5A**) [40]. Furthermore, we show that the RNAi phenotype is not due to off-target effects since we observe rescue on co-expression of a full-length *Gemin3* transgene. The possibility that this result is due to GAL4 dilution effects arising from multiple UAS-constructs in the background can be excluded since no rescue was observed when a truncated Gemin3 transgene (*Gem3* ^Δ*C*^) was utilised. Interestingly, we observed that although expression of the two RNAi transgenic constructs in the brain via the pan-neuronal *elav*-GAL4 or *n-syb*-GAL4 drivers had negligible effects on adult viability when development was restricted to a temperature of 25°C, a significant reduction (close to 50%) in adult viability was achieved when flies were cultured at a temperature of 29°C to allow for maximal GAL4 activity (**FIG. 5A**). Furthermore, we show that when the efficiency of knockdown was intensified further by increasing Dicer-2 levels, we achieved a dramatic reduction in adult viability or total lethality (**FIG. 5A**). Interestingly, we could repeat this result when Gemin3 knockdown is restricted exclusively to motor neurons via the *OK6*-GAL4 driver (**FIG. 5A**), a result that indicates a requirement of Gemin3 for viability specifically in motor neurons. Importantly, we show that similar to that observed when restricted to muscle (above), enhanced Gemin3 knockdown explicitly within the CNS leads to age-dependent progressive flight defects (**FIG. 5B**) and loss of adult viability (day 5, 100%; day 15, 100%; day 25, 72%; and, day 35, 26%) at an incubation temperature of 25°C. This finding indicates that in addition to muscle, sufficient Gemin3 levels in neurons are paramount for normal motor behaviour.

### Adequate Muscle and Neuronal Levels of Gemin5 are Required for Viability

**Figure 6:**
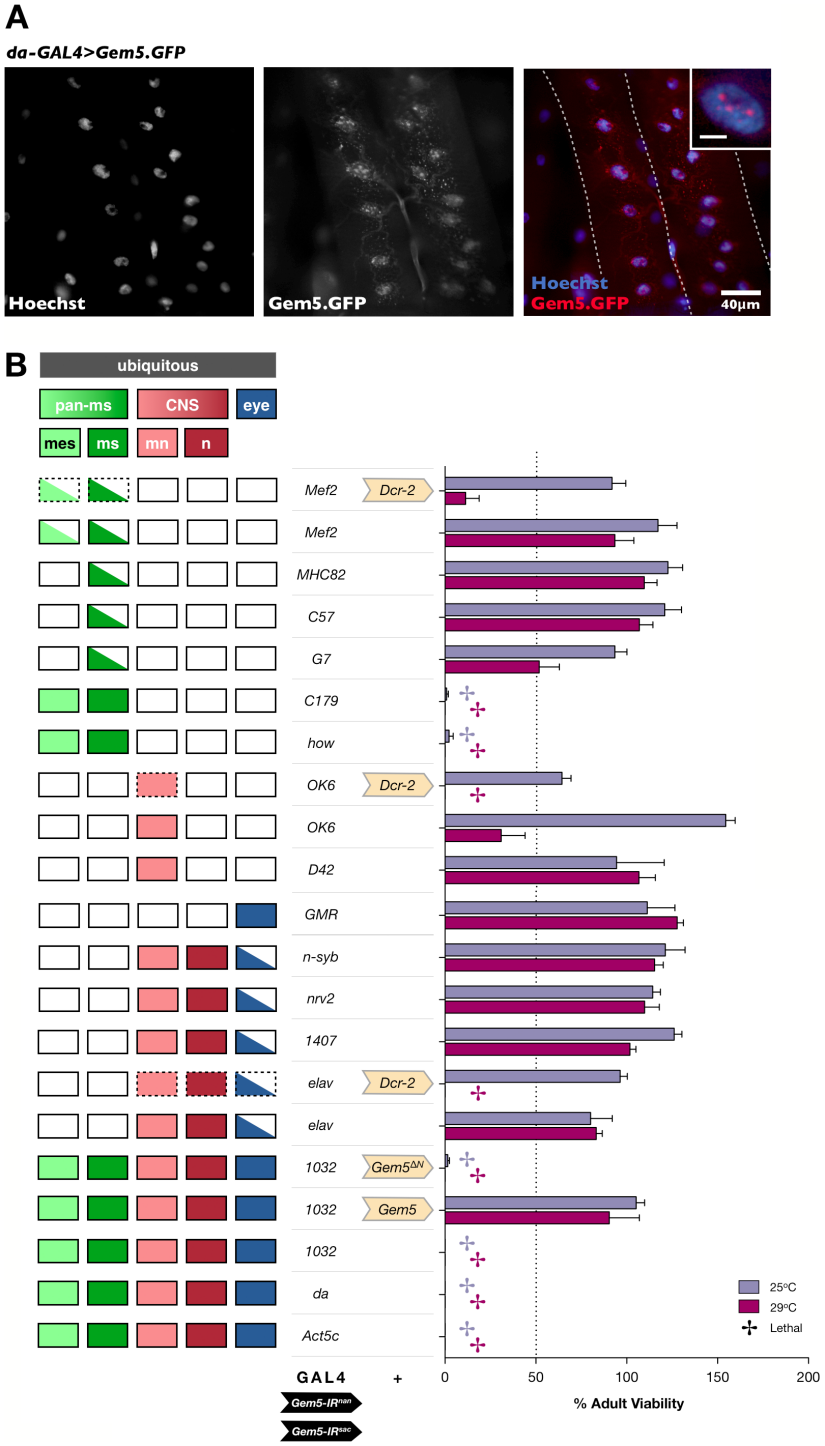
Gemin5 concentrates in predominantly nuclear foci and adequate levels in muscle as well as neurons are required for viability. (A) Larval muscles (delineated by dashed white outline) of flies ubiquitously expressing a GFP-tagged Gemin5 functional fusion protein. Gemin5 concentrates in puncta of variable size that are predominantly nuclear (insert, scale bar = 5 µm). For reasons as yet unclear, GFP is also detected within the tracheal system that supplies the muscles with oxygen. (B) A severe Gemin5 knockdown through the concomitant expression of two RNAi transgenes (*Gem5-IR^nan^+Gem5-IR^sac^*) targeting Gemin5 mRNA transcripts causes lethality when their expression is driven ubiquitously via the *1032*-GAL4, *da*-GAL4 or *Act5c*-GAL4 drivers. RNAi is specific since the co-expression of a full-length Gemin5 transgene (*Gem5*) but not a truncated version lacking its N-terminus (*Gem5* ^Δ*N*^) restores viability. Ubiquitous-associated lethality can be replicated if Gemin5 depletion is restricted to muscle lineages via the *C179*-GAL4 or *how*-GAL4 driver. A drastic reduction in viability can be observed when RNAi is driven via the pan-muscular *Mef2*-GAL4 driver at a temperature of 29°C and in the presence of enhanced Dicer-2 levels, both strategies employed to heighten the knockdown effect. Reduced levels of Gemin5 within the somatic musculature via the *G7*-GAL4 driver also had a marked negative impact on adult viability. A lethal outcome could also be obtained when RNAi is constrained to all CNS neurons (*elav*-GAL4) or exclusively to motor neurons (*OK6*-GAL4) at a temperature of 29°C and on upregulation of Dicer-2. Compared to controls, enhanced knockdown of Gemin5 in the eyes via the *GMR*-GAL4 driver does not lead to a rough eye phenotype (data not shown). Note that, as expected, not all drivers with a similar tissue expression pattern (left panel) give an analogous result with one reason being that they might not drive strong GAL4 levels comparable to their effective counterparts. Bar chart (right panel) shows adult fly viability assayed at 25°C and 29°C. Individual bars represent the mean viability ± S.E.M. of at least 4 independent experiments. Abbreviations: *pan-ms*, pan-muscular; *mes*, mesoderm; *ms*, larval muscles; *mn*, motor neurons; *n*, all CNS neurons except motor neurons.

We recently showed that as a member of the SMN complex, the *Drosophila* orthologue of Gemin5 (previously named Rigor mortis [61]) localises to U bodies within the ovarian cytoplasm [25]. Whilst our findings are indicative of a role for Gemin5 in an SMN complex-related activity, Kroiss et al. [39] argued otherwise based on a phylogenetic analysis revealing that *Drosophila* Gemin5 is evolving significantly faster compared to its orthologues in other organisms as well as a study reporting a function of the then Rigor mortis in ecdysone signalling [61]. In this context, we wished to further clarify the function of Gemin5 and hypothesised that if it is functionally associated with the SMN complex, its loss of function would mimic the key phenotypic outcomes resulting from loss of Gemin3, which itself is an integral SMN complex member. We first noticed that overexpression of a GFP-tagged Gemin5 fusion protein gave a similar sub-cellular localisation pattern to that we previously reported for Gemin3 [23]. Indeed, Gemin5 forms multiple nuclear foci of variable size within the nucleus and several smaller counterparts in the cytoplasm (**FIG. 6A**). Depending on their cellular location, these structures are most likely gems or U bodies if present in the nucleus or cytoplasm, respectively. We next attempted at addressing the relative requirement of Gemin5 in the two most important elements of the motor unit and asked whether Gemin5 is required in both muscle and neuron in a similar manner to Gemin3.

Enhanced Gemin5 knockdown through the expression of two RNAi transgenes (*Gem5-IR^nan^+Gem5-IR^sac^*) targeting the first exon of the Gemin5 mRNA transcript (**FIG S1B**) confirmed a previous report demonstrating that a global reduction of Gemin5 function is lethal [61] (**FIG. 6A**). We provide evidence for the specificity of the RNAi-based knockdown by showing that co-expression of a full-length Gemin5 transgene (*Gem5*) but not a version lacking the WD domain-rich N-terminus (*Gem5* ^Δ*N*^) rescues the lethality associated with loss of Gemin5 function (**FIG. 6A**; **FIG S1B**). The entire WD repeat domain was found to be both necessary and sufficient for sequence-specific, high-affinity binding of Gemin5 to snRNAs [35]. Interestingly, the lethality associated with ubiquitous Gemin5 knockdown can be recapped when knockdown is restricted to muscle lineages via the pan-muscular *how*-GAL4 or *C179*-GAL4 drivers. Furthermore, a dramatic reduction in adult viability was observed at culture temperatures associated with maximal GAL4 activity (29°C) when RNAi is driven by the pan-muscular *Mef2*-GAL4 and boosted by elevated Dicer-2 levels (**FIG. 6B**). Reduction of Gemin5 only in larval somatic muscles via the *G7*-GAL4 also resulted in a significant decrease in adult viability at an incubation temperature of 29°C. Intriguingly, we show that a pan-neuronal Gemin5 reduction via the *elav*-GAL4 driver together with an increase in Dicer-2 levels translates in lethality when flies are cultured at 29°C, and, remarkably, this phenotype can be repeated when knockdown is restricted to only motor neurons via the *OK6*-GAL4 driver. In summary, these studies underscore that similar to Gemin3, SMN complex member Gemin5 forms nuclear as well as cytoplasmic foci and is required in adequate levels within muscle and neurons for viability.

### Loss of Gemin5 Function in Either Muscle or Neurons Impairs Normal Motor Behaviour

**Figure 7:**
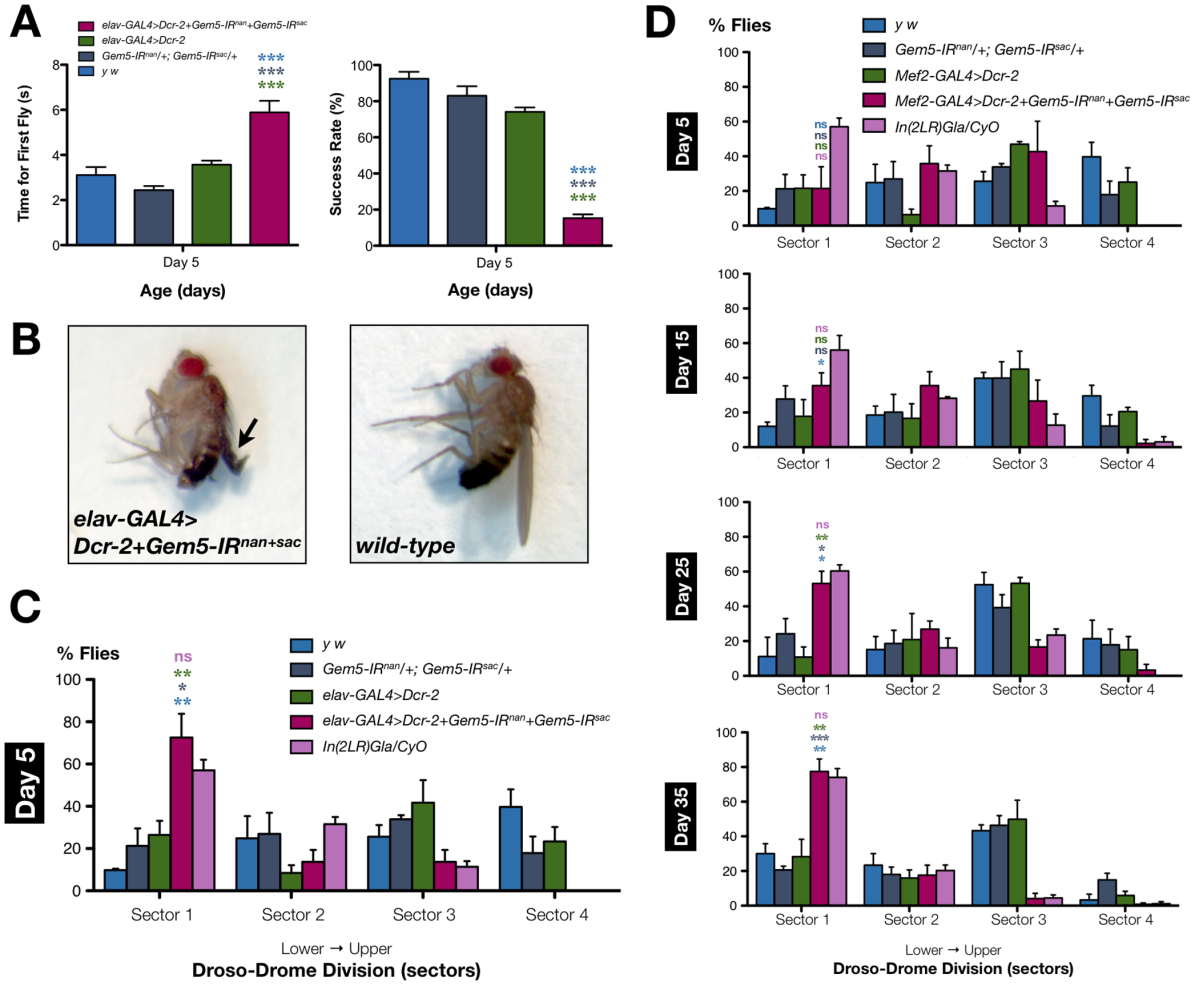
Loss of Gemin5 function in either neurons or muscle impedes normal motor behaviour. (**A**) Flies with a pan-neuronal Gemin5 knockdown (*elav-GAL4>Dcr-2+Gem5-IR^nan^+Gem5-IR^sac^*) have a significantly impaired climbing ability compared to wild-type (*y w*), RNAi transgenes-only (*Gem5-IR^nan^/+; Gem5-IR^sac^/+*) or driver-only (*elav-GAL4>Dcr-2*) controls, starting as early as day 5 post-eclosion. In this respect, the top performer in the population that had depleted levels of Gemin5 took significantly longer to climb beyond the 8 cm mark and on a population-level, the number of flies that successful reached a target height within the required timeframe was, on average, significantly lower compared to controls. (**B**) Compared to wild-type controls, close to half of the flies, with a CNS-restricted Gemin5 knockdown, had abnormal wing blades with defects ranging from a curved to a crumpled and unopened wing phenotype (arrow). (**C**) Flies with a brain-specific Gemin5 (*elav-GAL4>Dcr-2+Gem5-IR^nan^+Gem5-IR^sac^*) knockdown had on average a different distribution within the Droso-Drome compared to controls starting at day 5 post-eclosion. In this regard, the majority of flies drop straight to sector 1 indicating that they are flightless, a phenotypic outcome that differs significantly from that of controls but is similar to that of flies with curly wings (*In[2LR]Gla/CyO*), which are known to be weak fliers. (**D**) Pan-muscular Gemin5 knockdown leads to an age-dependent progressive decline in flight ability with the worse phenotype observed on day 35 post-eclosion. On this time-point, the majority of flies with muscle-specific depleted levels of Gemin5 are non-fliers and, thereby, fall straight to sector 1 in contrast to controls and similar to curly-positive flies. Data presented are the mean ± S.E.M. and statistical significance was determined for differences between the *elav-GAL4>Dcr-2+Gem5-IR^nan^+Gem5-IR^sac^* (A, C) or the *Mef2-GAL4>Dcr-2+Gem5-IR^nan^+Gem5-IR^sac^* (D) genotype and other genotypes, which are indicated by the respective colour. For all data, ns = not significant, *p<0.05, **p<0.01, and ***p<0.001.

Our findings indicating a requirement of Gemin5 for viability in the major constituents of the motor unit posed the question of whether loss of Gemin5 function in either muscle or neurons leads to defects in motor behaviour in a similar manner to Gemin3. In this regard, we first assessed the climbing ability of flies with an enhanced Gemin5 knockdown in the CNS, which are viable if their development is restricted to a permissive temperature (25°C). When we sampled flies with this genotype (*elav-GAL4>Dcr-2+Gem5-IR^nan^+Gem5-IR^sac^*), we noticed that the first fly that was capable of reaching the target height took, on average, significantly longer to do so compared to controls as early as day 5 post-eclosion. In the same vein, at this time point, brain-specific Gemin5 knockdown depressed markedly the climbing success rate or the average number of flies that successfully climbed beyond a target height of 8 cm by 10 seconds (**FIG. 7A**). Furthermore, we did not fail to observe that a small percentage of flies (36%) in this population had curved instead of normal straight wing blades and, less frequently (9%), the wings were crumpled and unopened (**FIG. 7B**).

The above results spurred us to investigate whether a drastic reduction of Gemin5 within the CNS has an adverse effect on flight performance. To this end, we subjected these flies to several Droso-Drome trials and compared the resulting mean to that of controls. Remarkably, the majority of flies with a pan-neuronal Gemin5 knockdown (*elav-GAL4>Dcr-2+Gem5-IR^nan^+Gem5-IR^sac^,* 73%*)* were flightless by day 5 post-eclosion, a result that is significantly different from that obtained by wild-type (*y w*, 10%), RNAi transgenes-only (*Gem5-IR^nan^/+; Gem5-IR^sac^/+,* 21%) or driver-only (*elav-GAL4>Dcr-2*, 26%) controls. However, the resulting phenotype did not differ from that of flies with a curly wing phenotype (*In[2LR]Gla/CyO*, 57%), which are known to have an impaired flight ability (**FIG. 7C**). We next asked whether restricting Gemin5 knockdown to muscle has a similar effect on motor behaviour. Although, a Dicer-boosted reduction in Gemin5 levels via the pan-muscular *Mef2*-GAL4 driver had no negative impact on climbing behaviour (data not shown), we demonstrate a striking progressive decline in flight ability throughout adulthood (**FIG. 7D**). Indeed, it is only on the final recorded time point (day 35 post-eclosion) that we get a majority of non-fliers (*Mef2-GAL4>Dcr-2+Gem5-IR^nan^+Gem5-IR^sac^*, 77%) in contrast to wild-type (*y w*, 30%), transgenic responder-only (*Gem5-IR^nan^/+; Gem5-IR^sac^/+*, 21%), or driver-only (*Mef2-GAL4>Dcr-2*, 28%) controls, and similar to the curly-positive flies (*In[2LR]Gla/CyO*, 74%). Overall, these findings indicate that loss of Gemin5 function in either muscle or neurons impairs normal motor behaviour, albeit with a different impact.

### Gemin2 is Required in Muscle for Viability and Normal Motor Behaviour

**Figure 8:**
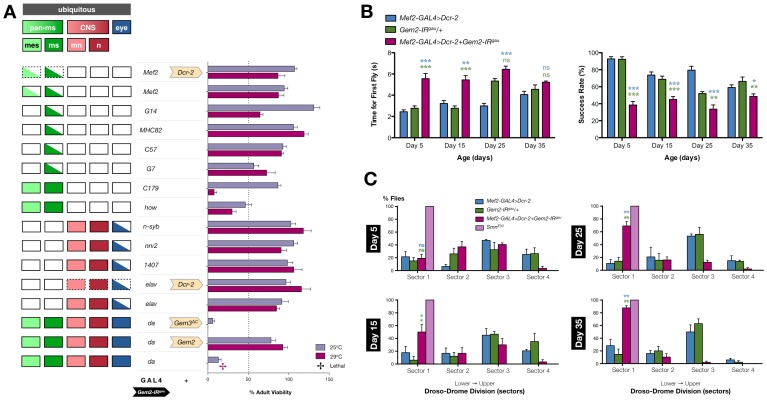
Gemin2 is essential for viability and normal motor function. (**A**) Gemin2 knockdown through the expression of a single RNAi transgene (*Gem2-IR^gau^*) causes a drastic reduction in adult viability (25°C) or complete lethality (29°C) if driven in all tissues via the *da*-GAL4 driver. Expression of transgenic *Gemin2* within this genetic background restores viability. No significant rescue is however observed on co-expression of an unrelated transgene (*Gem3* ^Δ*C*^). A marked reduction in adult viability can also be observed if Gemin2 is depleted specifically within muscle lineages (*C179*-GAL4 or *how*-GAL4). Note that, as expected, not all drivers with a similar tissue expression pattern (left panel) give an analogous result with one reason being that they might not drive strong GAL4 levels comparable to their effective counterparts. Bar chart (right panel) shows adult fly viability assayed at 25°C and 29°C, with the latter temperature inducing maximal GAL4 activity. Abbreviations: *pan-ms*, pan-muscular; *mes*, mesoderm; *ms*, larval muscles; *mn*, motor neurons; *n*, all CNS neurons except motor neurons. (**B**) Pan-muscular Gemin2 knockdown boosted by enhanced Dicer-2 levels leads to impaired climbing behaviour. In this respect, the time taken for the first fly within the sampled population to reach a target height of 8 cm is significantly increased on both day 5 and day 15 post-eclosion. In a similar vein, the climbing success rate or the percentage of flies that reach beyond 8 cm within 10 seconds, was dramatically decreased at all the recorded time points. (**C**) Flies with muscle-specific Gemin2 depletion exhibit a progressive age-dependent decline in flight performance. In this regard, the portion of flightless flies is significantly different from controls on day 15 post-eclosion and increases with age until reaching a maximal level on day 35 of adulthood. On the latter time point, the behaviour is similar to that of flies with reduced levels of SMN in muscle (*Smn^E33^*), which are 100% non-fliers. For all data, individual bars represent the mean ± S.E.M. and statistical significance was determined for differences between the *Mef2-GAL4>Dcr-2+Gem2-IR^gau^* genotype and control genotypes, which are indicated by the respective colour (B, C). For all data, ns = not significant, *p<0.05, **p<0.01, and ***p<0.001.

Gemin2 is the only SMN complex component with the most phylogenetically conserved sequence and domain structure [21], [33] (**FIG S4**). Its pivotal role in snRNP assembly has only recently been revealed through an elegant structural study [33]. It is however unclear whether this key SMN complex member is required for normal motor function. In this regard, double heterozygous *Gemin2* and *SMN* knockout mice develop an enhanced motor neurodegenerative phenotype, which correlated with disturbed snRNP assembly [62]. However, in zebrafish embryos, knockdown of Gemin2 was reported to have conflicting effects on motor axon outgrowth [63], [64], which is typically defective on depletion of SMN [65]. We sought to clarify this issue in *Drosophila* by targeting Gemin2 levels via the expression of an RNAi transgene (*Gem2-IR^gau^*) directed at exon 1 and part of exon 2 (**FIG S1C**). We note that similar to previous studies in other organisms [62], [64], [65], [66], global depletion of Gemin2 driven by the ubiquitous *da*-GAL4 driver results in lethality at culture temperatures that induce the highest GAL4 activity (**FIG. 8A**). Importantly, this phenotype is reversed on co-expression of a full-length Gemin2 transgene thereby demonstrating RNAi specificity. Furthermore, the lack of rescue by an unrelated truncated transgene (*Gem3* ^Δ*C*^) eliminates the possibility that rescue is the result of GAL4 dilution effects induced by multiple UAS-constructs in the background. Although Gemin2 knockdown was not maximal considering that we were limited by the use of a single RNAi transgene, we observed that on restricting its effect in various tissues, we could achieve a drastic reduction in adult viability through the use of the pan-muscular *C179*-GAL4 or *how*-GAL4 drivers (**FIG. 8A**).

Following up these observations, we asked whether Gemin2 knockdown in the motor unit has a negative impact on motor behaviour similar to that observed on depletion of either Gemin3 or Gemin5 (above). Interestingly, we show that when confined to muscle, Gemin2 reduction enhanced by high Dicer-2 levels interferes with mobility. In this respect, the time taken for the initial fly within the sampled population to cross a target height of 8 cm was significantly longer than that taken by controls at day 5 and day 15 post-eclosion. Furthermore, the climbing success rate is depressed as early as day 5 post-eclosion and remains at the same level throughout the course of adulthood (**FIG. 8B**). Notably, we demonstrate a progressive age-dependent decline in flight performance within the population of flies having a pan-muscular Gemin2 knockdown (*Mef2-GAL4>Dcr-2+Gem2-IR^gau^*) (**FIG. 8C**). In this regard, by day 15, half of the flies with this genotype were on average non-fliers (50%), a portion that was significantly different from that of the RNAi transgene-only (*Gem2-IR^gau^/+*, 6%) or driver-only (*Mef2-GAL4>Dcr-2*, 18%) controls. On the following two recorded time points, the fly population with a muscle-specific Gemin2 depletion, registered a further increase in the percentage of flies that are flightless. Indeed, the majority of flies (*Mef2-GAL4>Dcr-2+Gem2-IR^gau^*: day 25, 69%; day 35, 88%) drop straight to the lowest sector of the Droso-Drome in contrast to controls consisting of only the responder RNAi transgene (*Gem2-IR^gau^/+*: day 25, 14%; day 35, 15%) or driver (*Mef2-GAL4>Dcr-2*: day 25, 11%; day 35, 28%) and similar to the phenotypic outcome of flies homozygous for the hypomorphic *Smn^E33^* allele (**FIG. 8C**). Flies with a muscle-confined Gemin2 knockdown also experience a progressive decline in viability with age (day 5, 100%; day 15, 100%; day 25, 62%; and day 35, 47%). However, we did not detect significant motor defects when knockdown was restricted to the CNS (data not shown) but this outcome most likely reflects the fact that knockdown was not severe enough. In summary, these findings emphasise that similar to its counterparts within the SMN complex, Gemin2 is required in the motor unit for viability and normal motor function.

## Discussion

Our study sheds light on the antimorphic mechanism of the *Gem3* ^Δ*N*^ overexpression phenotype, and importantly unravels a requirement of all the Gemin associates of SMN in viability and motor function *in vivo*.

### Mechanism of the Gem3^ΔN^ Overexpression Phenotype

We demonstrate that the loss-of-function phenotype of Gemin3 is similar to that resulting from Gem3^ΔN^ overexpression. In this regard, either Gemin3 knockdown or Gem3^ΔN^ overexpression in muscle disrupt normal motor performance. Because overexpression mimics a loss of function, Gem3^ΔN^ presumably interferes at some level with the function of Gemin3, its complex or its RNA substrate, acting as a dominant-negative mutant or a Muller’s antimorph. It is fair to say that in the case of flight ability, phenotypes are not completely identical since those resulting from Gemin3 knockdown only develop late in adulthood. This observation highlights the fact that in contrast to RNAi-based methods, the use of inhibitory mutants that act at the protein level generate more severe phenotypes in view of them being more direct.

Several models can explain how overexpression of a catalytically inactive helicase protein antagonises the endogenous wild-type protein to exert a dominant-negative or antimorphic phenotype [46], [48]. Overexpression of a mutant RNA helicase protein that has defective ATPase or RNA unwinding activity but retains its ability to stably bind RNA may form a static protein-RNA complex that prevents RNA substrates from taking part in downstream reactions. This model is highly unlikely in our case considering that the Gem3^ΔN^ protein lacks its entire helicase core and, thereby, it most probably is unable to bind RNA, let alone engage in ATPase-dependent RNA chaperoning activities. Alternatively, a mutant dead helicase protein might have retained its ability to interact with its wild-type counterpart and the mutant/wild-type helicase dimer or multimer could be defective in its catalytic activity. Again, this model is highly improbable considering that to our knowledge the biochemical evidence to date supports an ability of SMN, Gemin2 and Gemin8 but not Gemin3 to self-associate [8], [9], [11], [31], [67]. This model is however the most probable in explaining the antimorphic mechanism of the hSMN overexpression phenotype ( [44] and present study). The resulting high hSMN levels are thought to titre dSMN into oligomers that might be unable to support the addition of the Gemin members of the SMN complex and, thereby, incapable of forming functional SMN complexes. Our demonstration of hSMN aggregates corroborates this mechanism though further work is needed to explore their toxic effect on cellular processes especially those confined to the nucleolus. In line with a previous study [60], muscle is more susceptible to attenuation of SMN activity, which in our case was induced by ectopic hSMN overexpression.

**Figure 9:**
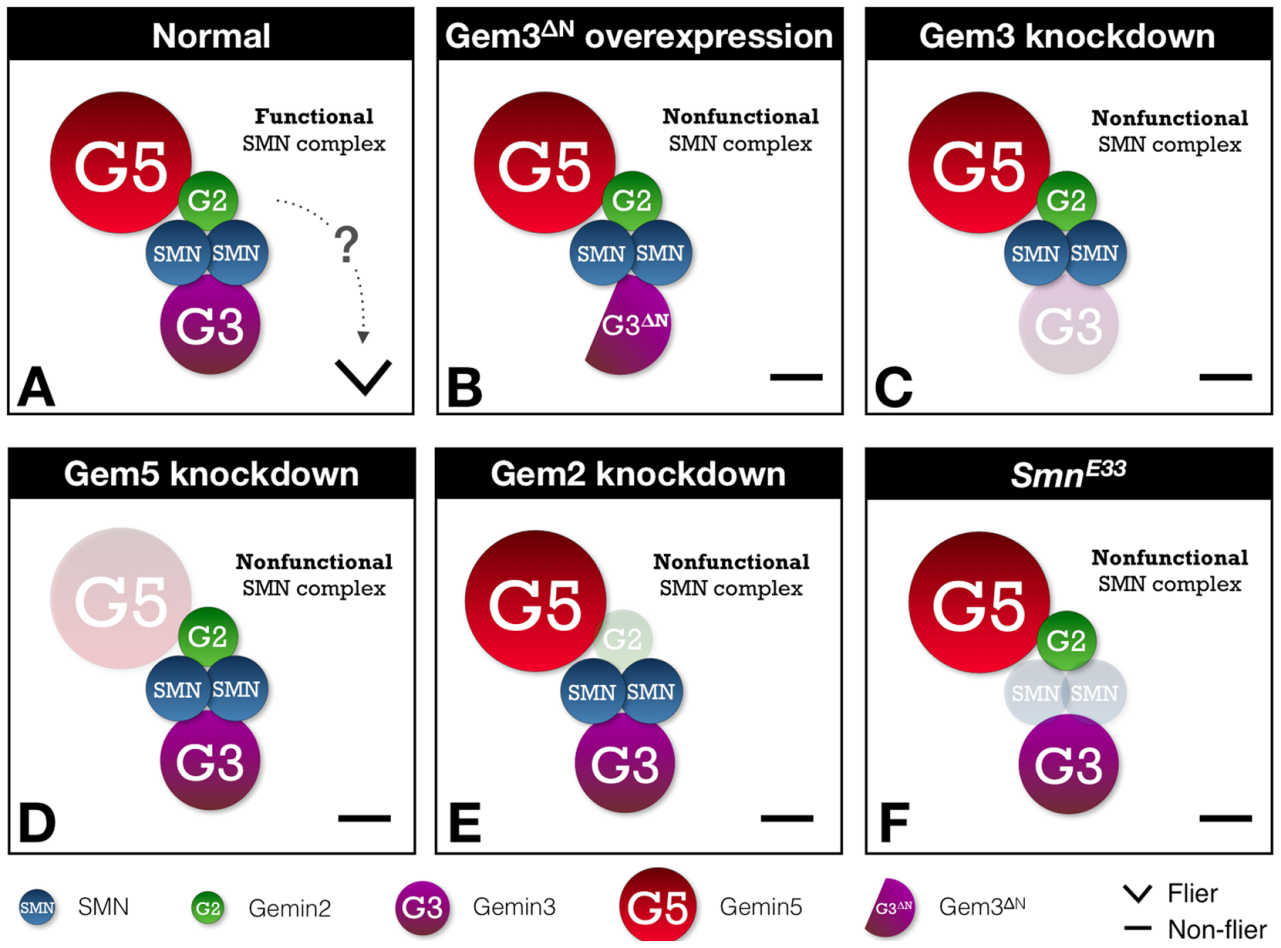
A working model for the evaluation of the motor phenotypes resulting from perturbation of individual members of the *Drosophila* SMN-Gemins complex. (A) The interaction map of the *Drosophila* SMN-Gemins complex is unclear and, here, it is modelled on that drafted for the human version (reviewed in [21]). In this regard, SMN associates to Gemin2 and Gemin3. Gemin2, in turn, interacts with Gemin5. All components of the SMN complex are individually required for viability and, importantly, motor behaviour. In particular, they are key for normal flight performance, which is a highly demanding motor activity that relies on correct function of the motor unit. The process or pathway linking the SMN complex to motor behaviour is as yet unclear (indicated by ‘?’) though we speculate that it is predominantly nuclear and, hence, might revolve around snRNP biogenesis and/or recycling. (B) The dead helicase Gem3^ΔN^ mutant lacks its helicase core but retains the SMN-binding domain. We hypothesise that it hijacks SMN and its associates from the endogenous wild-type Gemin3, hence, forming a non-functional or inactive SMN complex. Gem3^ΔN^ overexpression mimics the flightless phenotype associated with the loss of Gemin3 function though the phenotype is stronger. (C–E) Reduced Gemin3, Gemin5 or Gemin2 levels through RNAi-mediated knockdown also results in a flightless phenotype. These observations demonstrate that either of these factors performs a critical role that cannot be compensated by the presence of the remaining components. (D) Similar to its Gemin associates, SMN is required in adequate levels for normal flight behaviour as reported in the hypomorphic *Smn^E33^* fly mutant [43].

In another model, Gem3^ΔN^ may still be capable of binding to its partner and in high concentrations it competes that partner from the wild-type endogenous protein. This model is the most likely scenario in our case whereby the Gem3^ΔN^ protein, having retained its SMN-binding domain, hijacks SMN and its associates from the endogenous wild-type Gemin3, hence, forming a ‘poisonous’ or non-functional SMN complex (**FIG. 9A,B**). In this regard, although the SMN complex could still bind to target RNAs, it most probably is incapable of processing them due to loss of Gemin3 ATPase activity. This competition-based mechanism is supported by our observation that the Gem3^ΔN^ dominant-negative phenotype can be reversed by co-overexpression of the target protein, Gemin3, or aggravated in a background with reduced levels of Gemin3. Furthermore, the localisation pattern of the fluorescently-tagged version of Gem3^ΔN^ encourages us to hypothesise that its antagonistic action is primarily nuclear. It is possible that Gem3^ΔN^ causes mislocalisation of its associated complex from the cytoplasm to the nucleus, thereby displacing it away from its site of action. Nevertheless, it is still plausible that the smaller amount of Gem3^ΔN^ found in the cytoplasm could be sufficient to inhibit a cytoplasmic function. Interestingly, Almstead and Sarnow [68] reported that poliovirus-encoded proteinase 2A^pro^ specifically cleaves human Gemin3 between Tyr462 and Gly463 to generate a cleavage product that lacks its helicase core and, hence, is more or less identical to Gem3^ΔN^. Notably, poliovirus is the causative agent of poliomyelitis, which is characterised by destruction of motor neurons, a phenotype surprisingly similar to that observed in SMA. Future studies that focus on the interaction profile of Gem3^ΔN^ via biochemical approaches will help us in further understanding the molecular mode of action of this dead helicase mutant.

### Composition of the Drosophila SMN Complex

Throughout evolution, the SMN complex has experienced an increase in membership through the addition of a set of Gemin proteins onto an ancestral core complex formed of SMN and Gemin2 [21]. Focusing on *Drosophila*, Kroiss et al. [39] used a biochemical approach to successfully isolate an assembly-active SMN complex that surprisingly consisted of SMN and Gemin2 as the only stoichiometric components despite bioinformatic data that predicted the presence of Gemin3 and Gemin5 orthologues in this model organism. Later, we and others presented biochemical evidence showing interaction between Gemin3 and SMN [40], [49], [69]. In addition, the function of Gemin3 in snRNP assembly is conserved in *Drosophila* since, similar to that observed for SMN, depletion of Gemin3 significantly reduced Sm core assembly in an *in* *vitro* assay [49]. Such findings indicate that the *Drosophila* SMN complex is more intricate than that present in *S. pombe* and plants [21]. Taking into account *in* *vivo* work making use of genetic and cell biology approaches, we provide further support in this direction. The present study in combination with previous work on Gemin3 and SMN [40], [42], [43], [49], [60], unequivocally shows that remarkably similar phenotypes arise from loss of function of SMN and all the Gemin components including the less conserved Gemin3 and Gemin5 (**FIG S3** and **FIG. 9A,C–F**). This in itself is highly indicative of a common role for all the Gemins within the *Drosophila* SMN complex. Notably, our evidence is supported by a recent study in which we show that Gemins 2, 3 and 5 co-localise with SMN and snRNPs in cytoplasmic U bodies within the *Drosophila* egg chamber [25]. Furthermore, on overexpression of Gemin3 in *Drosophila*, we could induce the formation of SMN-enriched nuclear gems that interact with Cajal bodies in a similar manner to that described for their vertebrate counterparts [22], [23]. Interestingly, in this study we reported that gems could also be triggered by upregulation of Gemin5 protein levels. Although we cannot exclude the possibility of secondary SMN complex-independent activities, overall, the data captured so far, favours a *Drosophila* SMN complex formed of SMN, Gemin2, Gemin3 and Gemin5, all of which are essential for its correct functioning *in* *vivo*. Hence, we hypothesise that absence of any one member is sufficient to ‘rob’ the SMN-Gemins complex of its function (**FIG. 9A,C-F**). We are presently attempting genetic interaction studies to better understand the relationship between complex members *in* *vivo* and to strengthen our conviction that disruption of the SMN-Gemins complex is at the heart of the reported motor defects.

### A common Process that is key for Normal Motor Function

In this study we report that, depending on strength, loss of function of Gemin2, Gemin3 or Gemin5 in the motor unit impairs adult viability as well as normal motor behaviour including climbing ability and flight performance. In particular, flight is a highly demanding motor activity in flies and in addition to wings capable of generating enough aerodynamic lift, it depends on a motor-sensory circuit that allows exquisite control, and a high-power high-frequency muscle [53]. It is interesting to note that with the exception of Gemin2, which is required only in muscle, all Gemins seem to be required in both key compartments of the motor unit for survival and, importantly, motor function. In this regard, whether restricted to muscle or neurons, we showed that a strong loss of function of either Gemin3 or Gemin5 has a significant impact on adult viability whereas on milder knockdown, we could detect defects in motor function. A similar tissue-selective trend has been reported for SMN in various studies [42], [59], [60]. We also show that in corroboration with a previous study [49], forced expression of normal SMN in a Gem3^ΔN^ mutant background does not rescue the associated motor defects, thereby indicating that SMN does not function downstream of Gemin3 but most probably in parallel to Gemin3. Congruent with this, Gemins are not amongst the genetic modifiers of *Smn*-dependent phenotypes ([60], [70] and data not shown). Although a requirement of Gemin2 in neurons is as yet not obvious, we predict that, in a similar way to Gemin 3 and 5, such a role will be exposed only on significant knockdown. Hence, we are presently developing strategies aimed at increasing the impact of Gemin2 loss of function in neuronal tissues to test this hypothesis.

Key Gemin members of the SMN-Gemins complex have been reported to form part of additional multiprotein complexes in which they function independently of the SMN complex [21], [71]. For instance, Gemin3 and Gemin4 form a less abundant complex that co-sediments with polyribosomes and contains Argonaute2 (AGO2) as well as numerous microRNAs (miRNAs) [72], [73], [74]. In a following report, Gemin3 could be detected with AGO2, fragile X mental retardation protein (FMRP) and p100 in the murine peripheral axons of the sciatic nerve and all these proteins were reported to engage in the formation of an RNA-induced silencing complex (RISC) [75]. These studies implicate Gemin3 in RNA silencing, whereby aided by co-factors such as Gemin4, it may be responsible for RNA unwinding or RNP restructuring events during miRNA maturation and/or downstream events that include target RNA recognition. Furthermore, the association of Gemin3 with a multitude of transcription factors is well-documented [reviewed in 21], hence an additional role in transcriptional regulation cannot be excluded. An SMN complex-independent inclination was also reported for Gemin5, whereby it forms part of two distinct complexes, a specific internal ribosome entry site (IRES)-ribonucleoprotein complex and an IRES-independent complex containing eIF4E [76], [77], hence establishing a link between Gemin5 and modulation of mRNA translation. Turning to Gemin2, we note that there is evidence pointing towards the existence of an SMN-Gemin2 subcomplex that functions in DNA double-strand break repair through homologous recombination [78], [79]. This body of evidence seems to suggest alternative functions for some Gemin components, at least in vertebrates. However, based on our present work and that of others demonstrating a similar phenotype on loss of function of all members of the *Drosophila* SMN-Gemins complex [26], [27], [40], [42], [43], [49], [58], [60], [64], it is tempting to speculate that they engage in a common pathway or process *in* *vivo*.

The most-extensively studied pathway that unifies all members of the SMN complex is snRNP biogenesis. It is however still unclear how defects in this pathway are linked to impaired motor function. Recent studies on SMA animal models uncovered an negative impact on splicing and expression levels of U12 intron-containing genes that are essential for motor-sensory circuit function [80], [81] due to preferential reduction in the snRNPs that constitute the minor spliceosome [82], [83]. In this respect, an early study showed that the motor axon defects observed after silencing SMN and Gemin2 in zebrafish embryos could be rescued on injection of purified snRNPs [64] and recent work by Workman et al. [84] showed that restoring normal snRNP levels has a significant phenotypic rescuing effect on a severe SMA mouse model. The link between snRNP assembly and selective neuromuscular degeneration is however refuted by studies demonstrating that the role of SMN in snRNP assembly can be uncoupled from axonal defects in zebrafish [85] or organismal viability and locomotor defects in *Drosophila* [86]. Furthermore, there is evidence suggesting that splicing defects in SMA mice are likely a secondary consequence of severe SMN loss [83], [87]. Against this backdrop, the SMN complex has been implicated in two key non-canonical functions. In neuronal processes, the SMN complex is present in large stationary and small actively-transported granules devoid of Sm proteins [88], [89], [90], [91], [92]. On the postsynaptic side, the *Drosophila* SMN complex has been reported to localise to the sarcomeric Z-disc [43], [93]. In both compartments, the SMN complex might be involved in mRNA trafficking, a process required for the function and maintenance of neuromuscular junctions or myofibrils [21], [38]. Based on our evidence showing that localisation of the Gem3^ΔN^ mutant is predominantly nuclear coupled with the induction of nuclear gems following upregulation of Gemin3 or Gemin5, we find it tempting to speculate that the SMN-Gemins complex participates in a nucleocentric process or pathway that is key for normal motor function *in* *vivo*. We hypothesise that snRNP assembly and/or recycling are at the heart of this pathway, and studies that further our understanding of its components, its workings, and its manipulation through genetic and pharmacological means can potentially open up new avenues for SMA therapeutics.

## Materials and Methods

### Fly Stocks

Fly stocks were maintained at 25°C on standard molasses/maizemeal and agar medium in plastic vials. Except where indicated, the wild-type strains were *y w* or *Oregon R*. The *In(2LR)Gla/CyO* line was obtained from the *Drosophila* Genetic Resource Center (DGRC) at the Kyoto Institute of Technology, Kyoto, Japan. The *Smn^E33^* hypomorphic allele and *Smn^X7^* microdeletion were generous gifts from Greg Matera (University of North Carolina, Chapel Hill, North Carolina, USA) and Spyros Artavanis-Tsakonas (Harvard Medical School, Boston, Massachusetts, USA), respectively. The *UAS.SMN-YFP* and *UAS.hSMN* lines were kindly provided by Ji-Long Liu (MRC Functional Genomics Unit, University of Oxford, Oxford, UK). The *UAS.Gem3* ^ΔN^ transgenic construct as well as the RNAi transgenic constructs, *UAS.Gem3-IR^dwejra^* and *UAS.Gem3-IR^munxar^*, were characterised previously [40]. The RNAi transgenic constructs, *UAS.Gem2-IR^gaulos^* (10419R-1), *UAS.Gem5-IR^nanni^* (11171R-2), and *UAS.Gem5-IR^sacher^* (11171R-1), were obtained from the National Institute of Genetics Fly Stock Center, Japan.

GAL4 lines used in this work to drive expression of UAS-linked transgenes included *1032*-GAL4, *da*-GAL4, *α-Tub*-GAL4 (DGRC), *elav*-GAL4 (gift from Aaron Voigt, University Medical Center, RWTH Aachen, Germany), *elav-*GAL4*;UAS.Dcr-2* (Bloomington *Drosophila* Stock Centre [BDSC] at Indiana University, USA), *1407*-GAL4 (BDSC), *nrv2*-GAL4 (gift from Paul Salvaterra, City of Hope National Medical Center, Duarte, California, USA), *n-syb*-GAL4 (gift from Brian McCabe, Columbia University, NY, USA), *GMR*-GAL4 (DGRC), *D42*-GAL4 (BDSC), *OK6*-GAL4 (gift from Cahir O’Kane, University of Cambridge, Cambridge, UK), *C179*-GAL4 (BDSC), *how*-GAL4 (BDSC), *Mef2*-GAL4 (gift from Barry Dickson, Research Institute of Molecular Pathology, Vienna, Austria), *UAS.Dcr-2;Mef2-*GAL4 (BDSC), *G7*-GAL4 (gift from Aaron DiAntonio, Washington University, St. Louis, Missouri, USA), *C57*-GAL4 (gift from Vivian Budnik, University of Massachusetts, Worcester, Massachusetts, USA), and *MHC82*-GAL4 (gift from Cahir O’Kane). The spatial and temporal expression patterns are described in the Results section.

The generation of *UAS.Dcr-2;OK6-*GAL4, *UAS.Gem3-IR^dwe^;UAS.Gem3-IR^mun^*, *UAS.Gem5-IR^nan^;UAS.Gem5-IR^sac^* double transgenic stocks was carried out according to standard genetic crossing schemes.

### Transgenic Constructs

The generation of the *UAS.GFP.Gem3* ^Δ*N*^ transgenic construct involved PCR-amplification of the C-terminus of *Gemin3* and ligation, at the N-terminus, to the enhanced cyan fluorescent protein (eCFP) coding portion derived from the pECFP-C1 (BD Biosciences Clontech, Palo Alto, California, USA) vector. The *Not*I and *Xba*I restriction sites were then used to insert the fusion construct into the pUAST vector. For the *UAS.Gem3* ^Δ*C*^ transgenic construct, the N-terminus of *Gemin3* was PCR-amplified and ligated into the *Kpn*I and *Xba*I restriction sites of the pUAST vector. The *UAS.Gem2* transgenic construct involved PCR-amplification of the full-length coding sequence of *Gemin2* followed by insertion into the pUAST vector utilising the *Not*I and *Kpn*I restriction sites. PCR-amplification of the full-length coding sequence of Gemin5 or its C-terminus, followed by insertion into the *EcoR*I and *Kpn*I restriction sites of the pUAST vector, was carried out to generate the respective *UAS.Gem5.GFP* and *UAS.Gem5* ^Δ*N*^ transgenic constructs, with the former involving ligation of an eCFP tag at the C-terminus.

cDNA clones for Gemin2 (LD47479), Gemin3 (LD05563) and Gemin5 (SD03652) were obtained from the *Drosophila* Genomics Resource Centre (Indiana University, USA). In all cases, the ligation products were used to transform NEB 5-alpha competent *E. coli* cells (New England Biolabs, Hitchin, UK) using standard protocols. Correct transformants were further propagated, their harbouring plasmids purified and sequenced prior to microinjection in *w^1118^* embryos.

### Puparial Axial Ratios

Puparial axial ratios were calculated by dividing the length by the width of the puparia, both of which were measured from still images.

### Adult Viability Studies

Adult viability assays were conducted by crossing GAL4 driver stocks to lines harbouring single or a combination of transgenes. Culture temperature was either 25°C or 29°C. Following eclosion, adult flies were screened and counted at regular intervals. Adult viability was calculated as the percentage of the number of adult flies with the appropriate genotype divided by the expected number for the cross.

### Flight Assay

Based on that originally designed by the behavioural genetics mastermind, Seymour Benzer [56], we developed the Droso-Drome, a simple device to systematically measure the flying ability of flies. The Droso-Drome consisted of a 1L glass bottle divided into 4 sectors, of 5 cm each, spanning a total height of 20 cm. Before each assay, the internal walls of the Droso-Drome were coated with an alcohol-based sticky fluid. Flies were introduced into the top of the device through a funnel and the number of flies stuck in each sector were counted, divided by the total number of flies assayed and multiplied by 100 to generate the percentage number of flies per sector. The flight capability determines the height or sector flies stick in the Droso-Drome.

### Climbing Assay

The climbing apparatus consisted of two empty polystyrene vials that are vertically joined by tape facing each other. For the lower vial, a vertical distance of 8 cm above the bottom surface was measured and marked by drawing a circle around the entire circumference of the vial. For the climbing assay, a group of 10-15 flies were transferred into the lower vial and allowed to acclimatize to the new setting for 1 minute. The climbing assay involved gently tapping the flies down to the bottom of the vial and measuring (1) the number of flies per group, that can climb above the 8 cm mark by 10 seconds after the tap, recorded as the percentage success rate, and (2) the time for the first fly within a group to cross the 8 cm mark. Three trials were performed for each group and a minimum of three groups were assayed for each genotype. Experiments were performed during daylight to minimize potential effects of circadian oscillation.

### Immunohistochemistry

Larvae were dissected in 1× PBS, fixed in 4% paraformaldehyde in PBS and then washed in 1× PBS +0.1% Triton® X-100 (PBT). The tissues were next subjected to overnight staining at room temperature by mouse anti-GFP (1∶1000; Roche Diagnostics Ltd.) and mouse anti-hSMN 11F3 (1∶20; generous gift from Glenn Morris, Wolfson Centre for Inherited Neuromuscular Disease, RJAH Orthopaedic Hospital, Oswestry, UK) [94] antibodies. The next day, tissues were washed in PBT and stained overnight at room temperature with anti-mouse Alexa Fluor 546-conjugated secondary goat antibodies (1∶50) and nuclear-staining Hoechst 33342 (1∶500). Following a final wash in PBT, the samples were mounted in 90% glycerol with anti-fade. Epifluorescent pictures were acquired with an Optika B-600TiFL microscope (20× or 40× objectives).

### Statistical methods

Significance was tested by the unpaired t-test.

## Supporting Information

Figure S1 **Features of the transgenic constructs utilised in the study.** (**A**) *Gemin3* is a 4 Kbp two-exon gene with a set of nine DEAD-box helicase motifs present on its N-terminus and an SMN-binding region located in the middle. The *Gem3* ^Δ*N*^ construct is devoid of the N-terminal region, which hosts the helicase core but it still has the SMN-binding [11], [49] region. On the other hand, the *Gem3* ^Δ*C*^ is devoid of the C-terminal region, hence, consisting only of the helicase core. The inducible RNAi constructs targeting *Gemin3* (*Gem3-IR^mun^* and *Gem3-IR^dwe^*) both have a short fragment (highlighted in lavender) derived from exon1 and exon2 as an inverted repeat (IR), which is attached to 10 copies of UAS sites to enhance RNAi efficiency [51]. (**B**) *Gemin5* is a 6.8 Kbp nine-exon gene with thirteen WD repeat domains located at its N-terminus. The *Gem5* ^Δ*N*^ construct lacks the N-terminus and, hence, the region harbouring the snRNA-binding [35] WD-repeat domains. *Gemin5* mRNA transcripts were targeted by two inducible RNAi constructs (*Gem5-IR^nan^* and *Gem5-IR^sac^*), each consisting of an inverted repeat of a small fragment present in exon1 (highlighted in lavender). A Ret oncogene fragment (exon 5 to 7 including intron) is present between the IR fragments, thereby enhancing RNAi efficiency. (**C**) *Gemin2* is a 1.2 Kbp two-exon gene, which is highly conserved but lacks computationally identifiable domains. Both N- and C-terminus contain SMN- and self (G2)-binding domains [31]. Furthermore, crystal studies showed that SmF/E and SmD1/D1 make contact with Gemin2’s N-terminal and C-terminal domains, respectively [33]. Knockdown of Gemin2 was achieved through the expression of an inducible RNAi construct (*Gem2-IR^gau^*) consisting of the entire exon1 as well as part of exon2 in an inverted repeat that is also separated by a Ret oncogene fragment to boost RNAi efficiency.
(TIFF)

Figure S2 **Pan-muscular overexpression of hSMN has no effect on flight behaviour.** The distribution of organisms with a pan-muscular overexpression of hSMN (*Mef2-GAL4>hSMN*) was not significantly different from that of control populations (*Mef2-GAL4/+* or *hSMN/+*) over the course of 35 days post-eclosion. Note that on the final time point (day 35), the performance of the test genotype is significantly different from that of the driver-only control (*Mef2-GAL4/+*) but not the responder-only (*hSMN/+*) control. Data presented are the mean ± S.E.M. and statistical significance was determined for the differences, at sector 1, between the *Mef2-GAL4>hSMN* genotype and control genotypes, which are indicated by the respective colour. For all data, ns = not significant, *p<0.05, **p<0.01, and ***p<0.001.
(TIFF)

Figure S3 **Multiple protein sequence alignment of Gemin5 orthologues.** The protein sequence alignment was generated with the ClustalW at EMBL-EBI [95], [96] and displayed using GeneDoc (http://www.nrbsc.org/gfx/genedoc/). Human, *Homo sapiens* (Ensembl Protein ID: ENST00000285873); Mouse, *Mus musculus* (ENSMUST00000172035); Zebrafish, *Danio rerio* (ENSDART00000137309); Fly, *Drosophila melanogaster* (FBtr0086252). Conservation of sequence is represented based on the Gonnet Protein Weight Matrix, whereby conserved residues are shown in light grey (weakly conserved) to black (highly conserved). The N-terminus hosts the WD repeats (highlighted in red) and a coiled-coil motif (highlighted in blue) is present in the C-terminus.
(TIFF)

Figure S4 **Multiple protein sequence alignment of Gemin2 orthologues.** The protein sequence alignment was generated with the ClustalW at EMBL-EBI [95], [96] and displayed using GeneDoc (http://www.nrbsc.org/gfx/genedoc/). Human, *Homo sapiens* (Ensembl Protein ID: ENST00000308317); Mouse, *Mus musculus* (ENSMUST00000021379); Zebrafish, *Danio rerio* (ENSDART00000149779); Fly, *Drosophila melanogaster* (FBtr0075032). Conservation of sequence is represented based on the Gonnet Protein Weight Matrix, whereby conserved residues are shown in light grey (weakly conserved) to black (highly conserved).
(TIFF)

## References

[1] Kolb SJ, Sutton S, Schoenberg DR (2010) RNA processing defects associated with diseases of the motor neuron. Muscle Nerve 41: 5–17. 19697368 10.1002/mus.21428 PMC3654835

[2] Baumer D, Ansorge O, Almeida M, Talbot K (2010) The role of RNA processing in the pathogenesis of motor neuron degeneration. Expert reviews in molecular medicine 12: e21. 20642879 10.1017/S1462399410001523

[3] Lemmens R, Moore MJ, Al-Chalabi A, Brown RH Jr, Robberecht W (2010) RNA metabolism and the pathogenesis of motor neuron diseases. Trends in neurosciences 33: 249–258. 20227117 10.1016/j.tins.2010.02.003

[4] Sheean RK, Turner BJ (2013) Genetics of Motor Neuron Disorders: from gene diversity to common cellular conspirators in selective neuronal killing. In: Cauchi RJ, editor. Drosophila melanogaster Models of Motor Neuron Disease. N.Y.: Nova Biomedical. 1–34.

[5] Kolb SJ, Kissel JT (2011) Spinal muscular atrophy: a timely review. Archives of neurology 68: 979–984. 21482919 10.1001/archneurol.2011.74 PMC3860273

[6] Burghes AH, Beattie CE (2009) Spinal muscular atrophy: why do low levels of survival motor neuron protein make motor neurons sick? Nat Rev Neurosci 10: 597–609. 19584893 10.1038/nrn2670 PMC2853768

[7] Pellizzoni L, Charroux B, Dreyfuss G (1999) SMN mutants of spinal muscular atrophy patients are defective in binding to snRNP proteins. Proc Natl Acad Sci U S A 96: 11167–11172. 10500148 10.1073/pnas.96.20.11167 PMC18005

[8] Lorson CL, Strasswimmer J, Yao JM, Baleja JD, Hahnen E, et al (1998) SMN oligomerization defect correlates with spinal muscular atrophy severity. Nat Genet 19: 63–66. 9590291 10.1038/ng0598-63

[9] Young PJ, Man NT, Lorson CL, Le TT, Androphy EJ, et al (2000) The exon 2b region of the spinal muscular atrophy protein, SMN, is involved in self-association and SIP1 binding. Hum Mol Genet 9: 2869–2877. 11092763 10.1093/hmg/9.19.2869

[10] Liu Q, Fischer U, Wang F, Dreyfuss G (1997) The spinal muscular atrophy disease gene product, SMN, and its associated protein SIP1 are in a complex with spliceosomal snRNP proteins. Cell 90: 1013–1021. 9323129 10.1016/s0092-8674(00)80367-0

[11] Charroux B, Pellizzoni L, Perkinson RA, Shevchenko A, Mann M, et al (1999) Gemin3: a novel DEAD box protein that interacts with SMN, the spinal muscular atrophy gene product, and is a component of Gems. J Cell Biol 147: 1181–1193. 10601333 10.1083/jcb.147.6.1181 PMC2168095

[12] Charroux B, Pellizzoni L, Perkinson RA, Yong J, Shevchenko A, et al (2000) Gemin4: a novel component of the SMN complex that is found in both gems and nucleoli. J Cell Biol 148: 1177–1186. 10725331 10.1083/jcb.148.6.1177 PMC2174312

[13] Gubitz AK, Mourelatos Z, Abel L, Rappsilber J, Mann M, et al (2002) Gemin5, a novel WD repeat protein component of the SMN complex that binds Sm proteins. J Biol Chem 277: 5631–5636. 11714716 10.1074/jbc.M109448200

[14] Pellizzoni L, Baccon J, Rappsilber J, Mann M, Dreyfuss G (2002) Purification of native survival of motor neurons complexes and identification of Gemin6 as a novel component. J Biol Chem 277: 7540–7545. 11748230 10.1074/jbc.M110141200

[15] Baccon J, Pellizzoni L, Rappsilber J, Mann M, Dreyfuss G (2002) Identification and characterization of Gemin7, a novel component of the survival of motor neuron complex. J Biol Chem 277: 31957–31962. 12065586 10.1074/jbc.M203478200

[16] Carissimi C, Saieva L, Baccon J, Chiarella P, Maiolica A, et al (2006) Gemin8 is a novel component of the survival motor neuron complex and functions in small nuclear ribonucleoprotein assembly. J Biol Chem 281: 8126–8134. 16434402 10.1074/jbc.M512243200

[17] Carissimi C, Saieva L, Gabanella F, Pellizzoni L (2006) Gemin8 is required for the architecture and function of the survival motor neuron complex. J Biol Chem 281: 37009–37016. 17023415 10.1074/jbc.M607505200

[18] Campbell L, Unter KMD, Mohaghegh P, Tinsley JM, Brash MA, et al (2000) Direct interaction of Smn with dp103, a putative RNA helicase: role for Smn in transcription regulation? Hum Mol Genet 9: 1093–1100. 10767334 10.1093/hmg/9.7.1093

[19] Carissimi C, Baccon J, Straccia M, Chiarella P, Maiolica A, et al (2005) Unrip is a component of SMN complexes active in snRNP assembly. FEBS Lett 579: 2348–2354. 15848170 10.1016/j.febslet.2005.03.034

[20] Grimmler M, Otter S, Peter C, Muller F, Chari A, et al (2005) Unrip, a factor implicated in cap-independent translation, associates with the cytosolic SMN complex and influences its intracellular localization. Hum Mol Genet 14: 3099–3111. 16159890 10.1093/hmg/ddi343

[21] Cauchi RJ (2010) SMN and Gemins: ‘we are family’ … or are we? Insights into the partnership between Gemins and the spinal muscular atrophy disease protein SMN. Bioessays 32: 1077–1089. 20954180 10.1002/bies.201000088

[22] Liu Q, Dreyfuss G (1996) A novel nuclear structure containing the survival of motor neurons protein. Embo J 15: 3555–3565. 8670859 PMC451956

[23] Cauchi RJ (2011) Gem formation upon constitutive Gemin3 overexpression in Drosophila. Cell Biol Int 35: 1233–1238. 21627586 10.1042/CBI20110147

[24] Liu JL, Gall JG (2007) U bodies are cytoplasmic structures that contain uridine-rich small nuclear ribonucleoproteins and associate with P bodies. Proc Natl Acad Sci U S A 104: 11655–11659. 17595295 10.1073/pnas.0704977104 PMC1899408

[25] Cauchi RJ, Sanchez-Pulido L, Liu JL (2010) Drosophila SMN complex proteins Gemin2, Gemin3, and Gemin5 are components of U bodies. Exp Cell Res 316: 2354–2364. 20452345 10.1016/j.yexcr.2010.05.001

[26] Lee L, Davies SE, Liu JL (2009) The spinal muscular atrophy protein SMN affects Drosophila germline nuclear organization through the U body-P body pathway. Dev Biol 332: 142–155. 19464282 10.1016/j.ydbio.2009.05.553

[27] Cauchi RJ (2012) Conserved requirement for DEAD-box RNA helicase Gemin3 in Drosophila oogenesis. BMC Res Notes 5: 120. 22361416 10.1186/1756-0500-5-120 PMC3392723

[28] Buckingham M, Liu JL (2011) U bodies respond to nutrient stress in Drosophila. Experimental cell research 317: 2835–2844. 21939654 10.1016/j.yexcr.2011.09.001

[29] Workman E, Kolb SJ, Battle DJ (2012) Spliceosomal small nuclear ribonucleoprotein biogenesis defects and motor neuron selectivity in spinal muscular atrophy. Brain research 1462: 93–99. 22424789 10.1016/j.brainres.2012.02.051 PMC3448484

[30] Shpargel KB, Matera G (2005) Gemin proteins are required for efficient assembly of Sm-class ribonucleoproteins. Proc Natl Acad USA 102: 17372–17377. 10.1073/pnas.0508947102 PMC1297697 16301532

[31] Ogawa C, Usui K, Aoki M, Ito F, Itoh M, et al (2007) Gemin2 plays an important role in stabilizing the survival of motor neuron complex. J Biol Chem 282: 11122–11134. 17308308 10.1074/jbc.M609297200

[32] Feng W, Gubitz AK, Wan L, Battle DJ, Dostie J, et al (2005) Gemins modulate the expression and activity of the SMN complex. Hum Mol Genet 14: 1605–1611. 15843395 10.1093/hmg/ddi168

[33] Zhang R, So BR, Li P, Yong J, Glisovic T, et al (2011) Structure of a key intermediate of the SMN complex reveals Gemin2’s crucial function in snRNP assembly. Cell 146: 384–395. 21816274 10.1016/j.cell.2011.06.043 PMC3160754

[34] Battle DJ, Lau CK, Wan L, Deng H, Lotti F, et al (2006) The Gemin5 protein of the SMN complex identifies snRNAs. Mol Cell 23: 273–279. 16857593 10.1016/j.molcel.2006.05.036

[35] Lau CK, Bachorik JL, Dreyfuss G (2009) Gemin5-snRNA interaction reveals an RNA binding function for WD repeat domains. Nat Struct Mol Biol 16: 486–491. 19377484 10.1038/nsmb.1584

[36] Yong J, Kasim M, Bachorik JL, Wan L, Dreyfuss G (2010) Gemin5 delivers snRNA precursors to the SMN complex for snRNP biogenesis. Mol Cell 38: 551–562. 20513430 10.1016/j.molcel.2010.03.014 PMC2901871

[37] Yan X, Mouillet JF, Ou Q, Sadovsky Y (2003) A novel domain within the DEAD-box protein DP103 is essential for transcriptional repression and helicase activity. Mol Cell Biol 23: 414–423. 12482992 10.1128/MCB.23.1.414-423.2003 PMC140651

[38] Fallini C, Bassell GJ, Rossoll W (2012) Spinal muscular atrophy: the role of SMN in axonal mRNA regulation. Brain research 1462: 81–92. 22330725 10.1016/j.brainres.2012.01.044 PMC3360984

[39] Kroiss M, Schultz J, Wiesner J, Chari A, Sickmann A, et al (2008) Evolution of an RNP assembly system: a minimal SMN complex facilitates formation of UsnRNPs in Drosophila melanogaster. Proc Natl Acad Sci U S A 105: 10045–10050. 18621711 10.1073/pnas.0802287105 PMC2481332

[40] Cauchi RJ, Davies KE, Liu JL (2008) A motor function for the DEAD-box RNA helicase, Gemin3, in Drosophila. PLoS Genet 4: e1000265. 19023405 10.1371/journal.pgen.1000265 PMC2577925

[41] Grice SJ, Sleigh JN, Liu JL, Sattelle DB (2011) Invertebrate models of spinal muscular atrophy: insights into mechanisms and potential therapeutics. BioEssays : news and reviews in molecular, cellular and developmental biology 33: 956–965. 10.1002/bies.201100082 22009672

[42] Chan YB, Miguel-Aliaga I, Franks C, Thomas N, Trulzsch B, et al (2003) Neuromuscular defects in a Drosophila survival motor neuron gene mutant. Hum Mol Genet 12: 1367–1376. 12783845 10.1093/hmg/ddg157

[43] Rajendra TK, Gonsalvez GB, Walker MP, Shpargel KB, Salz HK, et al (2007) A Drosophila melanogaster model of spinal muscular atrophy reveals a function for SMN in striated muscle. J Cell Biol 176: 831–841. 17353360 10.1083/jcb.200610053 PMC2064057

[44] Miguel-Aliaga I, Chan YB, Davies KE, van den Heuvel M (2000) Disruption of SMN function by ectopic expression of the human SMN gene in Drosophila. FEBS Lett 486: 99–102. 11113446 10.1016/s0014-5793(00)02243-2

[45] Muller HJ (1932) Further studies on the nature and causes of gene mutations. Proceedings of the 6th Internatioinal Congress on Genetics, Ithaca, NY: 213–255.

[46] Prelich G (2012) Gene overexpression: uses, mechanisms, and interpretation. Genetics 190: 841–854. 22419077 10.1534/genetics.111.136911 PMC3296252

[47] Herskowitz I (1987) Functional inactivation of genes by dominant negative mutations. Nature 329: 219–222. 2442619 10.1038/329219a0

[48] Wu Y, Brosh RM Jr (2010) Helicase-inactivating mutations as a basis for dominant negative phenotypes. Cell cycle 9: 4080–4090. 20980836 10.4161/cc.9.20.13667 PMC3055193

[49] Shpargel KB, Praveen K, Rajendra TK, Matera AG (2009) Gemin3 is an essential gene required for larval motor function and pupation in Drosophila. Mol Biol Cell 20: 90–101. 18923150 10.1091/mbc.E08-01-0024 PMC2613097

[50] Cauchi RJ, van den Heuvel M (2006) The fly as a model for neurodegenerative diseases: is it worth the jump? Neurodegener Dis 3: 338–356. 17192723 10.1159/000097303

[51] Dietzl G, Chen D, Schnorrer F, Su KC, Barinova Y, et al (2007) A genome-wide transgenic RNAi library for conditional gene inactivation in Drosophila. Nature 448: 151–156. 17625558 10.1038/nature05954

[52] Klowden MJ (2007) Physiological Systems in Insects. NY: Academic Press.

[53] Dickinson M (2006) Insect flight. Current biology : CB 16: R309–314. 16682333 10.1016/j.cub.2006.03.087

[54] Vigoreaux JO (2006) Molecular Basis of Muscle Structure. In: Sink H, editor. Muscle Development in Drosophila. Texas: Landes Bioscience.

[55] Taylor MV (2006) Comparison of Muscle Development in Drosophila and Vertebrates. In: Sink H, editor. Muslce Development in Drosophila. Texas: Landes Bioscience.

[56] Benzer S (1973) Genetic dissection of behavior. Sci Am 229: 24–37. 4202065 10.1038/scientificamerican1273-24

[57] Crivat G, Taraska JW (2012) Imaging proteins inside cells with fluorescent tags. Trends in biotechnology 30: 8–16. 21924508 10.1016/j.tibtech.2011.08.002 PMC3246539

[58] Grice SJ, Liu JL (2011) Survival motor neuron protein regulates stem cell division, proliferation, and differentiation in Drosophila. PLoS Genet 7: e1002030. 21490958 10.1371/journal.pgen.1002030 PMC3072375

[59] Timmerman C, Sanyal S (2012) Behavioral and electrophysiological outcomes of tissue-specific Smn knockdown in Drosophila melanogaster. Brain research 1489: 66–80. 23103409 10.1016/j.brainres.2012.10.035 PMC3501589

[60] Chang HC, Dimlich DN, Yokokura T, Mukherjee A, Kankel MW, et al (2008) Modeling spinal muscular atrophy in Drosophila. PLoS ONE 3: e3209. 18791638 10.1371/journal.pone.0003209 PMC2527655

[61] Gates J, Lam G, Ortiz JA, Losson R, Thummel CS (2004) rigor mortis encodes a novel nuclear receptor interacting protein required for ecdysone signaling during Drosophila larval development. Development 131: 25–36. 14645129 10.1242/dev.00920

[62] Jablonka S, Holtmann B, Meister G, Bandilla M, Rossoll W, et al (2002) Gene targeting of Gemin2 in mice reveals a correlation between defects in the biogenesis of U snRNPs and motoneuron cell death. Proc Natl Acad USA 99: 10126–10131. 10.1073/pnas.152318699 PMC126635 12091709

[63] McWhorter ML, Boon KL, Horan ES, Burghes AH, Beattie CE (2008) The SMN binding protein Gemin2 is not involved in motor axon outgrowth. Dev Neurobiol 68: 182–194. 18000835 10.1002/dneu.20582

[64] Winkler C, Eggert C, Gradl D, Meister G, Giegerich M, et al (2005) Reduced U snRNP assembly causes motor axon degeneration in an animal model for spinal muscular atrophy. Genes Dev 19: 2320–2330. 16204184 10.1101/gad.342005 PMC1240041

[65] McWhorter ML, Monani UR, Burghes AHM, Beattie CE (2003) Knockdown of the survival motor neuron (SMN) protein in zebrafish causes defects in motor axon outgrowth and pathfinding. J Cell Biol 162: 919–931. 12952942 10.1083/jcb.200303168 PMC1761110

[66] Burt EC, Towers PR, Sattelle DB (2006) Caenorhabditis elegans in the study of SMN-interacting proteins: a role for SMI-1, an orthologue of human Gemin2 and the identification of novel components of the SMN complex. Invert Neurosci 6: 145–159. 16964508 10.1007/s10158-006-0027-x

[67] Otter S, Grimmler M, Neuenkirchen N, Chari A, Sickmann A, et al (2007) A comprehensive interaction map of the human survival of motor neuron (SMN) complex. J Biol Chem 282: 5825–5833. 17178713 10.1074/jbc.M608528200

[68] Almstead LL, Sarnow P (2007) Inhibition of U snRNP assembly by a virus-encoded proteinase. Genes Dev 21: 1086–1097. 17473171 10.1101/gad.1535607 PMC1855234

[69] Guruharsha KG, Obar RA, Mintseris J, Aishwarya K, Krishnan RT, et al (2012) Drosophila protein interaction map (DPiM): a paradigm for metazoan protein complex interactions. Fly 6: 246–253. 23222005 10.4161/fly.22108 PMC3519659

[70] Sen A, Dimlich DN, Guruharsha KG, Kankel MW, Hori K, et al (2013) Genetic circuitry of Survival motor neuron, the gene underlying spinal muscular atrophy. Proceedings of the National Academy of Sciences of the United States of America 110: E2371–2380. 23757500 10.1073/pnas.1301738110 PMC3696827

[71] Battle DJ, Kasim M, Wang J, Dreyfuss G (2007) SMN-independent subunits of the SMN complex. Identification of a small nuclear ribonucleoprotein assembly intermediate. J Biol Chem 282: 27953–27959. 17640873 10.1074/jbc.M702317200

[72] Dostie J, Mourelatos Z, Yang M, Sharma A, Dreyfuss G (2003) Numerous microRNPs in neuronal cells containing novel microRNAs. Rna 9: 180–186. 12554860 10.1261/rna.2141503 PMC1370383

[73] Mourelatos Z, Dostie J, Paushkin S, Sharma A, Charroux B, et al (2002) miRNPs: a novel class of ribonucleoproteins containing numerous microRNAs. Genes Dev 16: 720–728. 11914277 10.1101/gad.974702 PMC155365

[74] Nelson PT, Hatzigeorgiou AG, Mourelatos Z (2004) miRNP:mRNA association in polyribosomes in a human neuronal cell line. Rna 10: 387–394. 14970384 10.1261/rna.5181104 PMC1370934

[75] Murashov AK, Chintalgattu V, Islamov RR, Lever TE, Pak ES, et al (2007) RNAi pathway is functional in peripheral nerve axons. Faseb J 21: 656–670. 17209129 10.1096/fj.06-6155com

[76] Pacheco A, Lopez de Quinto S, Ramajo J, Fernandez N, Martinez-Salas E (2009) A novel role for Gemin5 in mRNA translation. Nucleic Acids Res 37: 582–590. 19066202 10.1093/nar/gkn979 PMC2632916

[77] Fierro-Monti I, Mohammed S, Matthiesen R, Santoro R, Burns JS, et al (2006) Quantitative proteomics identifies Gemin5, a scaffolding protein involved in ribonucleoprotein assembly, as a novel partner for eukaryotic initiation factor 4E. J Proteome Res 5: 1367–1378. 16739988 10.1021/pr0504539

[78] Takizawa Y, Qing Y, Takaku M, Ishida T, Morozumi Y, et al.. (2010) GEMIN2 promotes accumulation of RAD51 at double-strand breaks in homologous recombination. Nucleic Acids Res. 10.1093/nar/gkq271 PMC2926616 20403813

[79] Takaku M, Tsujita T, Horikoshi N, Takizawa Y, Qing Y, et al (2011) Purification of the human SMN-GEMIN2 complex and assessment of its stimulation of RAD51-mediated DNA recombination reactions. Biochemistry 50: 6797–6805. 21732698 10.1021/bi200828g

[80] Lotti F, Imlach WL, Saieva L, Beck ES, Hao le T, et al (2012) An SMN-dependent U12 splicing event essential for motor circuit function. Cell 151: 440–454. 23063131 10.1016/j.cell.2012.09.012 PMC3474596

[81] Imlach WL, Beck ES, Choi BJ, Lotti F, Pellizzoni L, et al (2012) SMN is required for sensory-motor circuit function in Drosophila. Cell 151: 427–439. 23063130 10.1016/j.cell.2012.09.011 PMC3475188

[82] Gabanella F, Butchbach ME, Saieva L, Carissimi C, Burghes AH, et al (2007) Ribonucleoprotein Assembly Defects Correlate with Spinal Muscular Atrophy Severity and Preferentially Affect a Subset of Spliceosomal snRNPs. PLoS ONE 2: e921. 17895963 10.1371/journal.pone.0000921 PMC1976558

[83] Zhang Z, Lotti F, Dittmar K, Younis I, Wan L, et al (2008) SMN deficiency causes tissue-specific perturbations in the repertoire of snRNAs and widespread defects in splicing. Cell 133: 585–600. 18485868 10.1016/j.cell.2008.03.031 PMC2446403

[84] Workman E, Saieva L, Carrel TL, Crawford TO, Liu D, et al (2009) A SMN missense mutation complements SMN2 restoring snRNPs and rescuing SMA mice. Human molecular genetics 18: 2215–2229. 19329542 10.1093/hmg/ddp157 PMC2685758

[85] Carrel TL, McWhorter ML, Workman E, Zhang H, Wolstencroft EC, et al (2006) Survival motor neuron function in motor axons is independent of functions required for small nuclear ribonucleoprotein biogenesis. J Neurosci 26: 11014–11022. 17065443 10.1523/JNEUROSCI.1637-06.2006 PMC6674655

[86] Praveen K, Wen Y, Matera AG (2012) A Drosophila model of spinal muscular atrophy uncouples snRNP biogenesis functions of survival motor neuron from locomotion and viability defects. Cell reports 1: 624–631. 22813737 10.1016/j.celrep.2012.05.014 PMC3405901

[87] Baumer D, Lee S, Nicholson G, Davies JL, Parkinson NJ, et al (2009) Alternative splicing events are a late feature of pathology in a mouse model of spinal muscular atrophy. PLoS Genet 5: e1000773. 20019802 10.1371/journal.pgen.1000773 PMC2787017

[88] Zhang H, Xing L, Rossoll W, Wichterle H, Singer RH, et al (2006) Multiprotein complexes of the survival of motor neuron protein SMN with gemins traffic to neuronal processes and growth cones of motor neurons. J Neurosci 26: 8622–8632. 16914688 10.1523/JNEUROSCI.3967-05.2006 PMC4956918

[89] Todd AG, Morse R, Shaw DJ, Stebbings H, Young PJ (2010) Analysis of SMN-neurite granules: Core Cajal body components are absent from SMN-cytoplasmic complexes. Biochem Biophys Res Commun 397: 479–485. 20515655 10.1016/j.bbrc.2010.05.139

[90] Todd AG, Shaw DJ, Morse R, Stebbings H, Young PJ (2010) SMN and the Gemin proteins form sub-complexes that localise to both stationary and dynamic neurite granules. Biochemical and biophysical research communications 394: 211–216. 20188701 10.1016/j.bbrc.2010.02.158

[91] Fallini C, Zhang H, Su Y, Silani V, Singer RH, et al (2011) The survival of motor neuron (SMN) protein interacts with the mRNA-binding protein HuD and regulates localization of poly(A) mRNA in primary motor neuron axons. The Journal of neuroscience : the official journal of the Society for Neuroscience 31: 3914–3925. 21389246 10.1523/JNEUROSCI.3631-10.2011 PMC3070748

[92] Briese M, Esmaeili B, Sattelle DB (2005) Is spinal muscular atrophy the result of defects in motor neuron processes? Bioessays 27: 946–957. 16108074 10.1002/bies.20283

[93] Walker MP, Rajendra TK, Saieva L, Fuentes JL, Pellizzoni L, et al (2008) SMN complex localizes to the sarcomeric Z-disc and is a proteolytic target of calpain. Hum Mol Genet 17: 3399–3410. 18689355 10.1093/hmg/ddn234 PMC2566527

[94] Young PJ, Le TT, thi Man N, Burghes AH, Morris GE (2000) The relationship between SMN, the spinal muscular atrophy protein, and nuclear coiled bodies in differentiated tissues and cultured cells. Exp Cell Res 256: 365–374. 10772809 10.1006/excr.2000.4858

[95] Goujon M, McWilliam H, Li W, Valentin F, Squizzato S, et al (2010) A new bioinformatics analysis tools framework at EMBL-EBI. Nucleic acids research 38: W695–699. 20439314 10.1093/nar/gkq313 PMC2896090

[96] Larkin MA, Blackshields G, Brown NP, Chenna R, McGettigan PA, et al (2007) Clustal W and Clustal X version 2.0. Bioinformatics 23: 2947–2948. 17846036 10.1093/bioinformatics/btm404

