# Supplementary figures and images for: The Gemin Associates of Survival Motor Neuron Are Required for Motor Function in Drosophila
Source: PLoS One. 2013 Dec 31;8(12):e83878. doi: 10.1371/journal.pone.0083878 (PMC3877121; doi:10.1371/journal.pone.0083878)

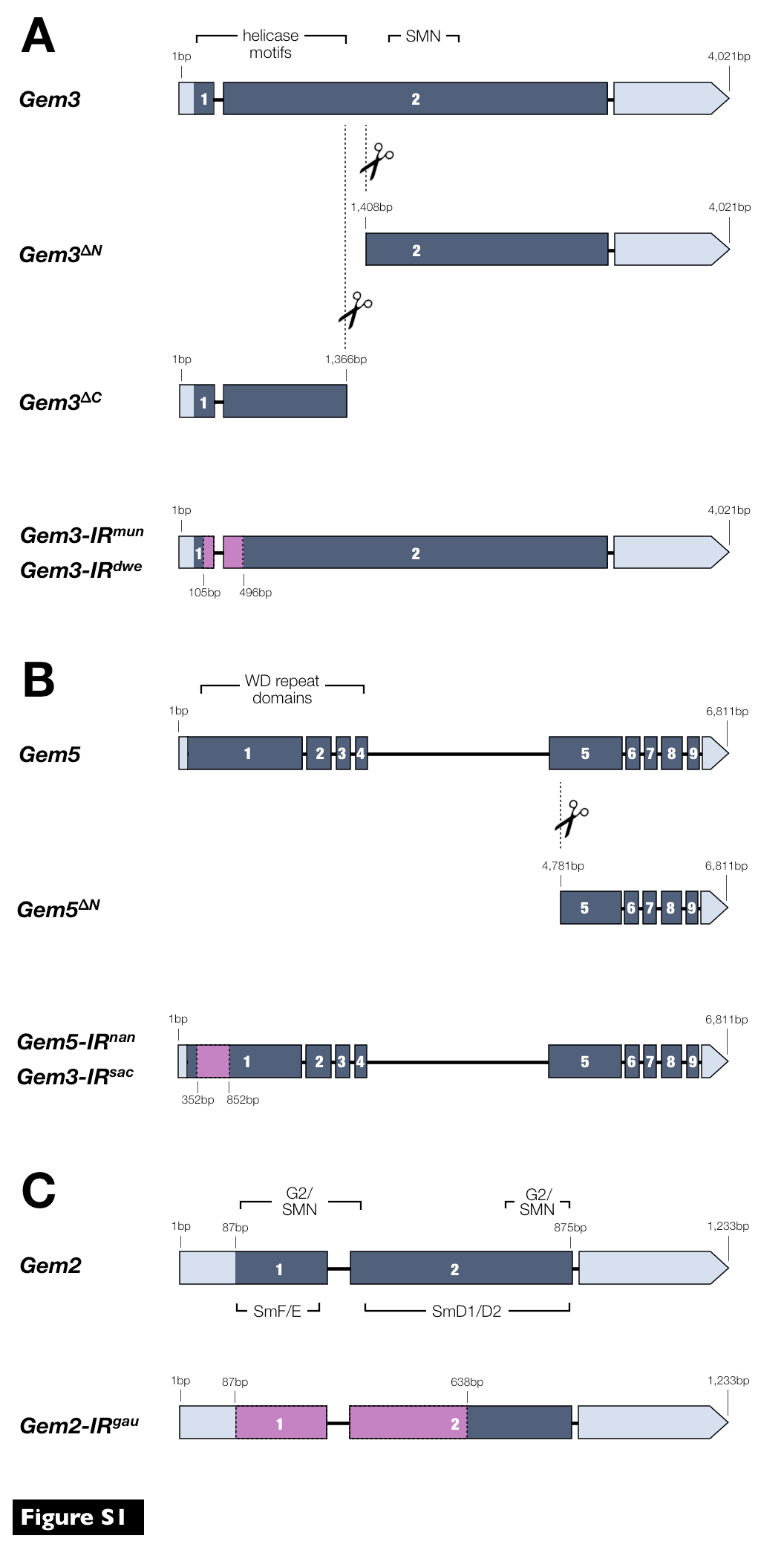

Supplement: Figure S1 — Features of the transgenic constructs utilised in the study. (A) Gemin3 is a 4 Kbp two-exon gene with a set of nine DEAD-box helicase motifs present on its N-terminus and an SMN-binding region located in the middle. The Gem3 ΔN construct is devoid of the N-terminal region, which hosts the helicase core but it still has the SMN-binding [11], [49] region. On the other hand, the Gem3 ΔC is devoid of the C-terminal region, hence, consisting only of the helicase core. The inducible RNAi constructs targeting Gemin3 (Gem3-IRmun and Gem3-IRdwe) both have a short fragment (highlighted in lavender) derived from exon1 and exon2 as an inverted repeat (IR), which is attached to 10 copies of UAS sites to enhance RNAi efficiency [51]. (B) Gemin5 is a 6.8 Kbp nine-exon gene with thirteen WD repeat domains located at its N-terminus. The Gem5 ΔN construct lacks the N-terminus and, hence, the region harbouring the snRNA-binding [35] WD-repeat domains. Gemin5 mRNA transcripts were targeted by two inducible RNAi constructs (Gem5-IRnan and Gem5-IRsac), each consisting of an inverted repeat of a small fragment present in exon1 (highlighted in lavender). A Ret oncogene fragment (exon 5 to 7 including intron) is present between the IR fragments, thereby enhancing RNAi efficiency. (C) Gemin2 is a 1.2 Kbp two-exon gene, which is highly conserved but lacks computationally identifiable domains. Both N- and C-terminus contain SMN- and self (G2)-binding domains [31]. Furthermore, crystal studies showed that SmF/E and SmD1/D1 make contact with Gemin2’s N-terminal and C-terminal domains, respectively [33]. Knockdown of Gemin2 was achieved through the expression of an inducible RNAi construct (Gem2-IRgau) consisting of the entire exon1 as well as part of exon2 in an inverted repeat that is also separated by a Ret oncogene fragment to boost RNAi efficiency. (TIFF) [file pone.0083878.s001.tiff]

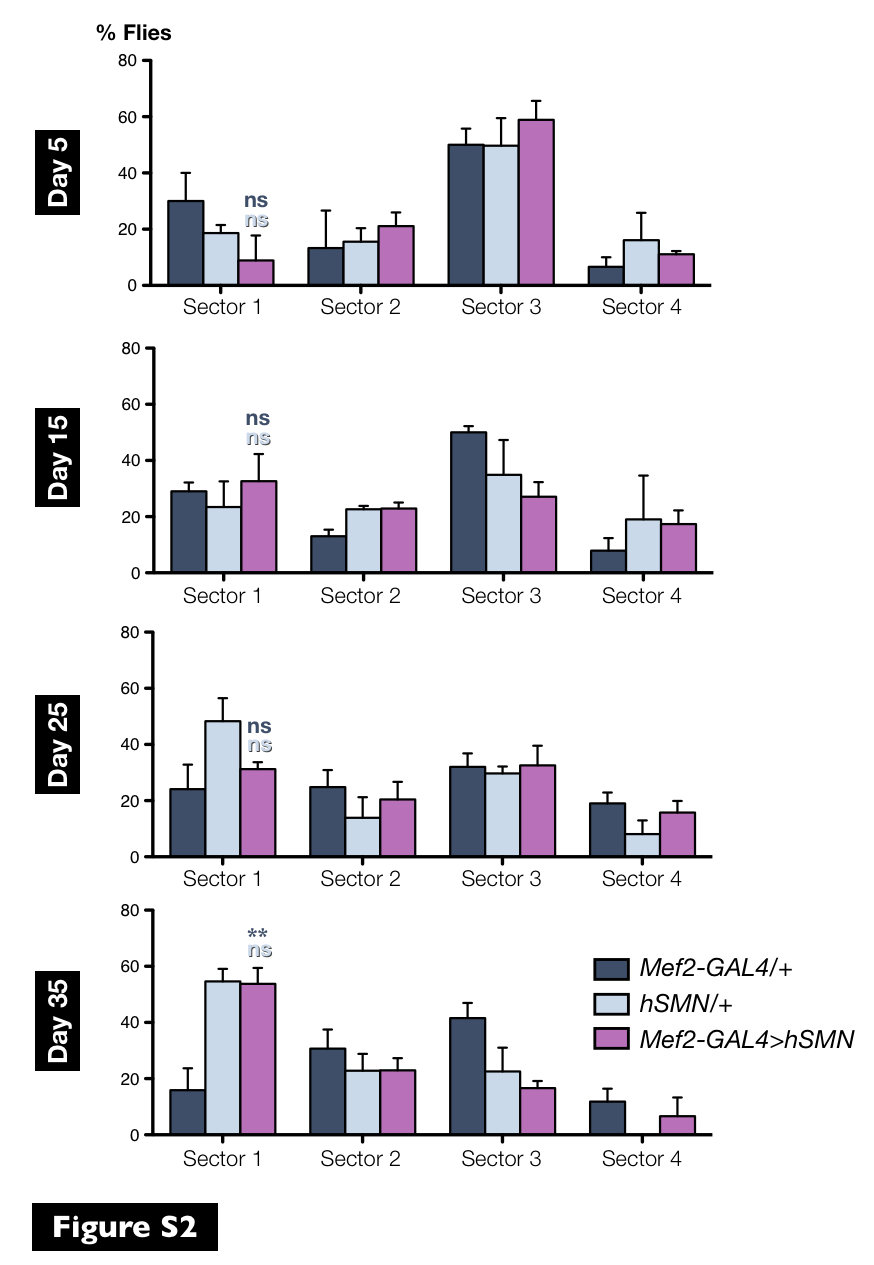

Supplement: Figure S2 — Pan-muscular overexpression of hSMN has no effect on flight behaviour. The distribution of organisms with a pan-muscular overexpression of hSMN (Mef2-GAL4>hSMN) was not significantly different from that of control populations (Mef2-GAL4/+ or hSMN/+) over the course of 35 days post-eclosion. Note that on the final time point (day 35), the performance of the test genotype is significantly different from that of the driver-only control (Mef2-GAL4/+) but not the responder-only (hSMN/+) control. Data presented are the mean ± S.E.M. and statistical significance was determined for the differences, at sector 1, between the Mef2-GAL4>hSMN genotype and control genotypes, which are indicated by the respective colour. For all data, ns = not significant, *p<0.05, **p<0.01, and ***p<0.001. (TIFF) [file pone.0083878.s002.tiff]

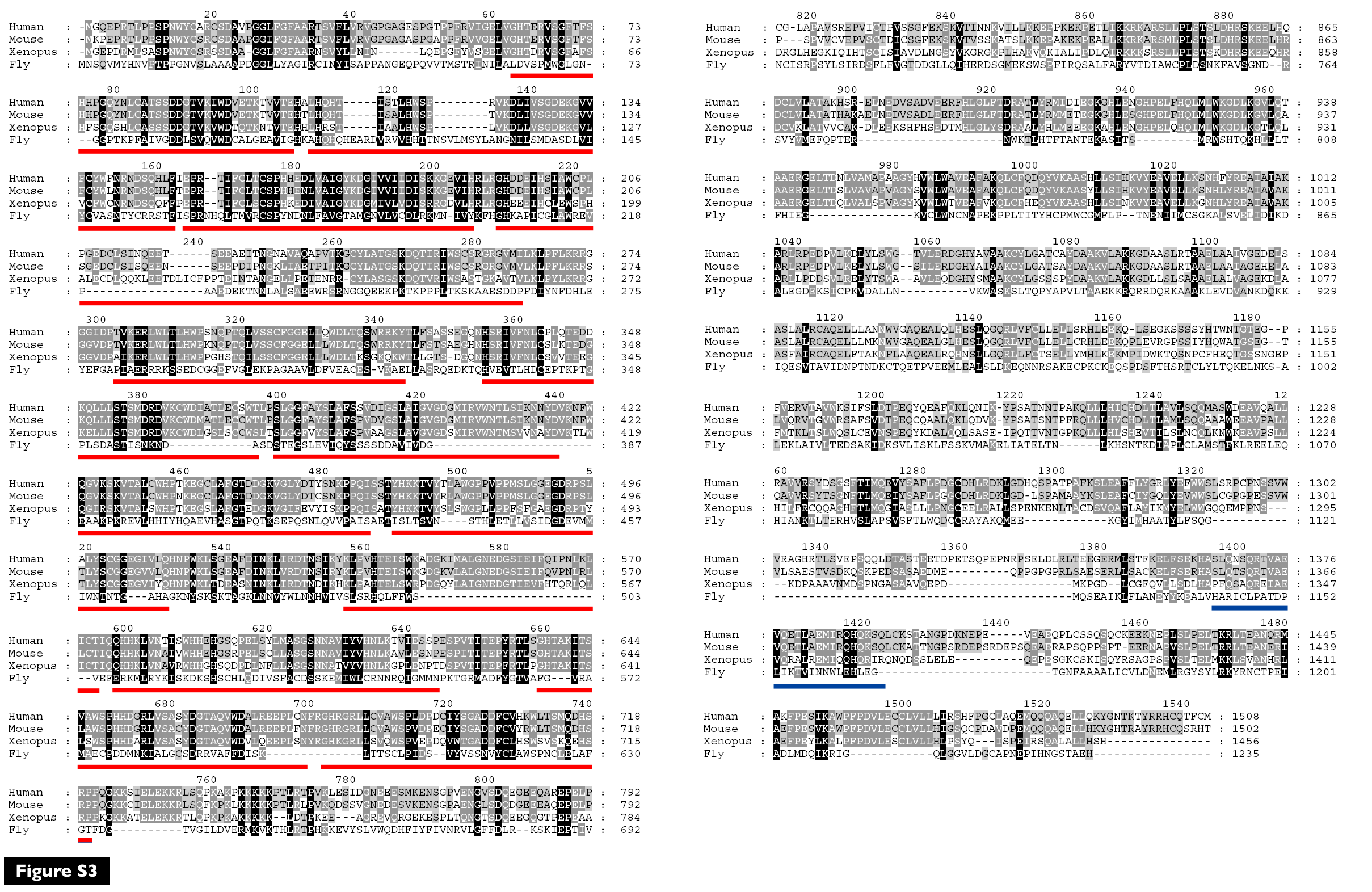

Supplement: Figure S3 — Multiple protein sequence alignment of Gemin5 orthologues. The protein sequence alignment was generated with the ClustalW at EMBL-EBI [95], [96] and displayed using GeneDoc (http://www.nrbsc.org/gfx/genedoc/). Human, Homo sapiens (Ensembl Protein ID: ENST00000285873); Mouse, Mus musculus (ENSMUST00000172035); Zebrafish, Danio rerio (ENSDART00000137309); Fly, Drosophila melanogaster (FBtr0086252). Conservation of sequence is represented based on the Gonnet Protein Weight Matrix, whereby conserved residues are shown in light grey (weakly conserved) to black (highly conserved). The N-terminus hosts the WD repeats (highlighted in red) and a coiled-coil motif (highlighted in blue) is present in the C-terminus. (TIFF) [file pone.0083878.s003.tiff]

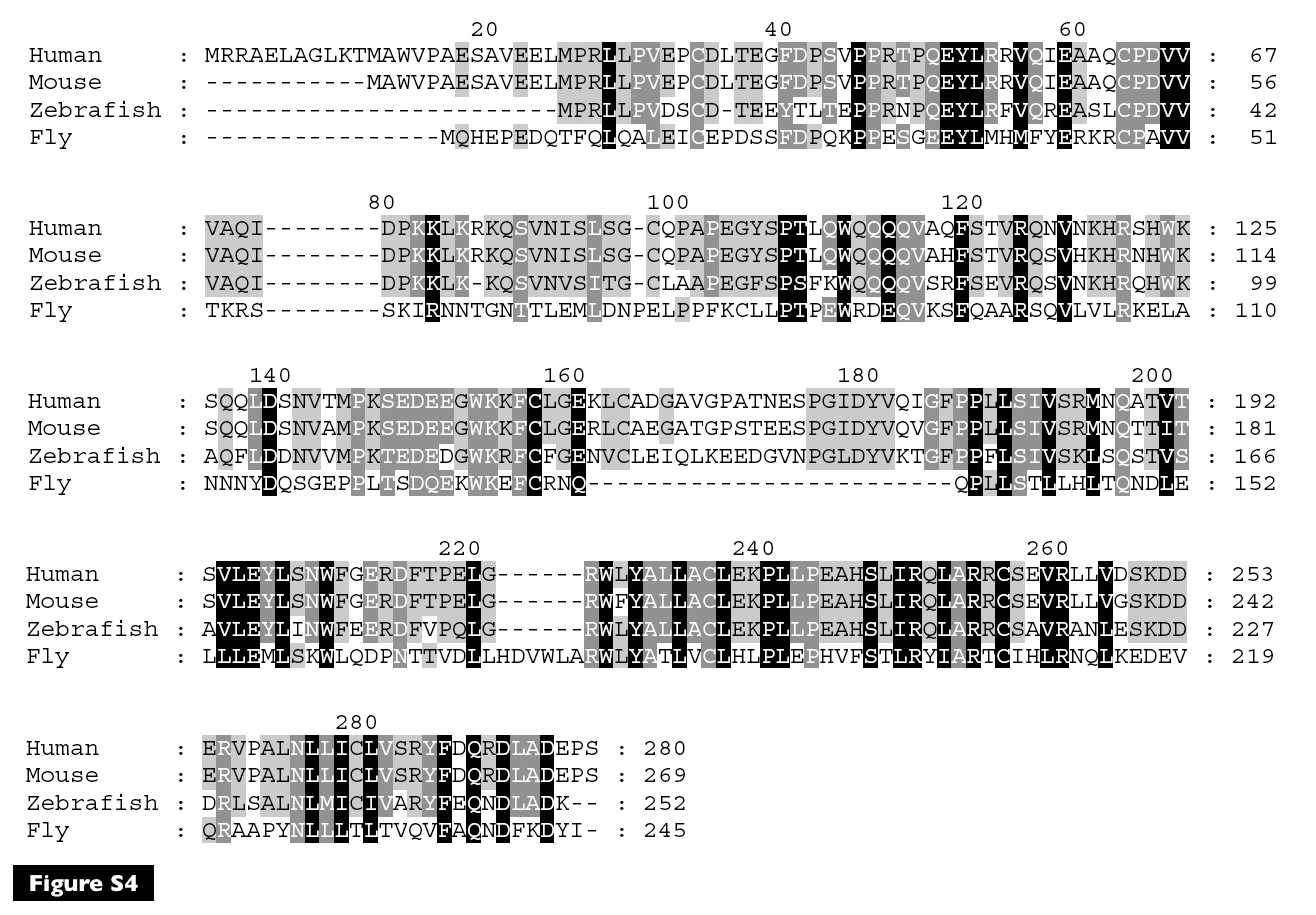

Supplement: Figure S4 — Multiple protein sequence alignment of Gemin2 orthologues. The protein sequence alignment was generated with the ClustalW at EMBL-EBI [95], [96] and displayed using GeneDoc (http://www.nrbsc.org/gfx/genedoc/). Human, Homo sapiens (Ensembl Protein ID: ENST00000308317); Mouse, Mus musculus (ENSMUST00000021379); Zebrafish, Danio rerio (ENSDART00000149779); Fly, Drosophila melanogaster (FBtr0075032). Conservation of sequence is represented based on the Gonnet Protein Weight Matrix, whereby conserved residues are shown in light grey (weakly conserved) to black (highly conserved). (TIFF) [file pone.0083878.s004.tiff]
